# Maximizing Insights, Minimizing Animal Testing: A Framework for Validating Multiparametric Single‐Cell Cytokine Analysis Panels

**DOI:** 10.1002/eji.202451193

**Published:** 2025-03-12

**Authors:** Johann Aleith, Wendy Bergmann‐Ewert, Brigitte Müller‐Hilke

**Affiliations:** ^1^ Core Facility for Cell Sorting and Cell Analysis Rostock University Medical Center Rostock Germany

**Keywords:** Brefeldin A, fibroblast‐like synoviocytes, helper T cells, immune cells, intracellular cytokine staining, spectral flow cytometry

## Abstract

Intracellular cytokine labeling combined with high‐parametric flow cytometry offers substantial promise in elucidating the nuanced effector functions of cells. However, the establishment of complex multicolor panels is often laborious and the importance of validation processes may be underestimated in research practice. This raises the risk of prematurely translating multicolor panels into in vivo studies. Alternatively, researchers may resort to animal disease models to procure cytokine‐producing cells. Both scenarios raise ethical concerns as they entail the potential for unnecessary animal suffering without yielding novel insights into immunobiology. Here, we perform multicolor panel optimization and validation without the need for stressful animal testing. We designed two spectral flow cytometry panels for cytokine expression analyses across mouse immune and joint cells. Animal testing was replaced by stimulated co‐cultures of T cells, splenocytes, and fibroblast‐like synoviocytes. These cultures were used for multicolor labeling experiments. Our method proved suitable for validating the two cytometry panels, as it provided a complex cellular environment in which a variety of cytokine‐producing populations were identified. In summary, we here present a blueprint for the quality control of single‐cell cytokine assays by cell culture and further introduce multicolor panels that can be employed for studies on inflammatory or infectious diseases.

## Graphical abstract

Multicolor spectral flow cytometry panels for cytokine expression analysis were optimized and validated without animal testing. Stimulated co‐cultures of helper T cells, splenocytes, and fibroblast‐like synoviocytes provided a complex cellular environment suitable for validating intracellular cytokine staining. This approach offers a more ethical alternative to commonly used animal disease models.

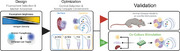

## 1 Introduction

Flow cytometry is a powerful tool for the investigation of biological systems, providing extensive insights into the phenotypic and functional characteristics of cells, both in health and disease. Its versatility and robustness have made it a pivotal method for hypothesis testing and exploratory experimentation, respectively, across multiple research areas, including immunology, pathology, and drug testing [1, 2, 3]. Notably, in the last few years, spectral flow cytometry emerged as a groundbreaking technical advancement, permitting the integration of an extensive array of fluorophores, thus enhancing the complexity of cellular analyses [4]. In detail, the ability of spectral flow cytometry to exploit off‐peak fluorescence signatures enables the incorporation and resolution of spectrally overlapping fluorophores with emission maxima at similar wavelengths [5]. Consequently, spectral flow cytometry facilitates flexible fluorophore‐to‐marker allocations (i.e., panel design) and allows exhaustive sample interrogation [5, 6]. By implementation of intracellular cytokine staining (ICS) in particular, modern single‐cell immunophenotyping assays are able to delineate effector function dynamics in low‐frequency immune cell populations [7]. These assays are conventionally performed after stimulating ex vivo cell cultures and treating them with protein transport inhibitors, such as Brefeldin A, which increase signal resolution by causing intracellular cytokine accumulation [7, 8]. Alternatively, ICS can be performed directly on cells obtained from test animals treated with Brefeldin A, enabling the interrogation of in situ cytokine production while preserving the physiological tissue environment [9].

With the ongoing technological progress in flow cytometry instrumentation, the establishment and validation of multicolor panels are increasingly challenging [10]. For example, optimizing these panels involves labor‐intensive and sometimes complicated processes. The effort, however, is worthwhile as enriching the scope of information extracted from individual samples fosters comprehensive insights into cellular dynamics, especially when sample material is limited [6, 11]. In the context of animal models, high‐dimensional data acquisition allows more thorough data collection for smaller sample sizes and thus holds promise for reducing the total number of animals required for experiments. Conversely, researchers often turn to in vivo disease models to obtain validation controls for single‐cell cytokine analysis, as healthy laboratory animals may lack cells with sufficient cytokine production [11, 12]. This practice not only amplifies the risk of causing undue animal suffering but also contributes to increased animal usage, contradicting the principles of the 3Rs [13]. Hence, in order to further reduce the amount of animal testing, a framework is warranted that replaces animal experiments with in vitro methods for the establishment of spectral flow cytometry panels that involve intracellular cytokines staining.

In this work, we designed flow cytometry panels for the study of mouse cytokine expressions among adaptive and innate immune cell populations, as well as stromal cells. These panels were specifically tailored to analyze cytokinogenesis in mouse models that incorporate in vivo Brefeldin A injection. Our main goal was to provide researchers with an easy‐to‐implement and resource‐effective protocol for establishing such panels while minimizing animal suffering. For this, mice were euthanized under full anesthesia, with no procedures performed that could induce distress, pain, or anxiety. Using primary mouse cells and simple in vitro differentiation and stimulation protocols, different concentrations of antibody‐fluorophore conjugates were tested for surface and intracellular antigens. In order to avoid the usage of animal disease models, multicolor panels were validated utilizing an easily adoptable co‐culture model capable of depicting cytokine expressions across a variety of cell subtypes. As of yet, only a small number of animal studies describe the in vivo application of Brefeldin A [9, 14, 15, 16, 17]. Hence, safety data for this compound and its use in small animals are still lacking. The information on whether Brefeldin A usage leads to animal harm is of great importance when applying to ethics committees for permission to carry out animal experiments. We therefore injected Brefeldin A into a small number of mice and analyzed for both, animal wellbeing and alterations of the immune landscape in various organs compared with mice that did not receive the injection. In summary, we here introduce a simple strategy to design in vitro validation controls for spectral flow cytometry panels that are applicable to (1) intracellular cytokine staining and (2) the study of animal models of autoimmunity and infection.

## 2 Results

### 2.1 Design and Optimization of Panels for Single‐Cell Cytokine Analysis Without Stressful Animal Testing

We here designed two different full spectrum flow cytometry panels which will allow us to differentiate common mouse immune cell lineages via both, surface antigen and ICS. In brief, we initially assessed fluorophore availability from various manufacturers and opted for commonly used dyes (e.g., FITC, PE, and APC) for markers with limited conjugation options (e.g., cytokines). Similarly, rarely available fluorophores were chosen for common antigens (e.g., APC/Fire810 for CD45). We simultaneously selected bright fluorophores for low‐density antigens (e.g., PE for CCL2), focusing on optimal resolution for cytokine labeling. In order to reduce spreading errors, we further assigned fluorophores with similar spectral signatures (e.g., BV480 and BV510) to antigens that are not usually co‐expressed (e.g., CD3 and B220). Due to the high autofluorescence exhibited by myeloid cells, we avoided combining markers recognizing these cells with dyes emitting at 450–540 nm, as autofluorescence peaks within this range [5, 18]. Finally, we refrained from using dim colors for antigens expressed on highly autofluorescent cells and instead allocated them to cells with minimal autofluorescence. Table S1 lists the antibody‐fluorophore combinations, a fixable dye for the discrimination of dead cells, and the final allocation of each fluorophore to its respective detector. In summary, Panel 1 included 20 antibody‐fluorophore conjugates for the identification of leukocytes (CD45), T lymphocytes (CD3, CD4, CD8, CD25), B lymphocytes (B220), natural killer (NK) cells (CD49b), myeloid cells (CD11b, CD11c, Gr‐1, F4/80), co‐stimulatory cells (CD80, CD86) as well as cytokines that are expressed by innate and adaptive immune cells, respectively (TNFα, IL‐6, CCL2, CCL3, IL‐10, IFNγ, IL‐17A). For Panel 2, we selected 23 conjugates that were mostly recycled from Panel 1, with the addition of markers for the identification of endothelial or fibroblast‐like cells (CD31, CD54, CD90, and CD106). Due to the limited commercial availability of suitable antigen‐fluorophore combinations, we excluded CD49b from Panel 2. Thus, while Panel 1 incorporates markers for a more general approach to immunophenotyping and serves as a scaffold for more specialized assays, Panel 2 expands on this by incorporating specific markers that address joint cells.

In order to enable the testing of different antibody concentrations for multicolor staining and to simultaneously evaluate the labeling quality for each antibody‐fluorophore conjugate, we utilized either freshly isolated or cultured primary mouse cells. Figure S1 illustrates the cell types that were employed for single antibody staining of surface antigens. Figures S1 and S2 show the distribution of fluorescent cells for each marker as well as the respective detector's background fluorescence. For the review of constitutively expressed immune cell markers, we simply used splenocytes and gated either on FSC^lo^ cells for lymphoid lineage antigens or on FSC^hi^ cells for myeloid lineage antigens (Figures S1A, S2A). Heat‐killed splenocytes were used in order to robustly stain dead cells (Figure S2A). We expected that only a small percentage of splenocytes would express CD25 and therefore used a culture of activated CD4^+^ lymph node cells (Figure S1B). We further found that CD80 was expressed at a low density on splenocytes (data not shown) and hence used bone marrow‐derived macrophages (BMDMs) instead. Besides a robust expression of F4/80, BMDMs were indeed effectively labeled for CD80 (Figure S1C). For the assessment of CD31 labeling, we enzymatically digested small intestines and identified a distinct population of CD31^+^ endothelial cells (Figure S1D). We further isolated and cultured fibroblast‐like synoviocytes (FLS) from mouse paws and successfully labeled them for CD54, CD90, and CD106 (Figures S1E, S2A).

**FIGURE 1:**
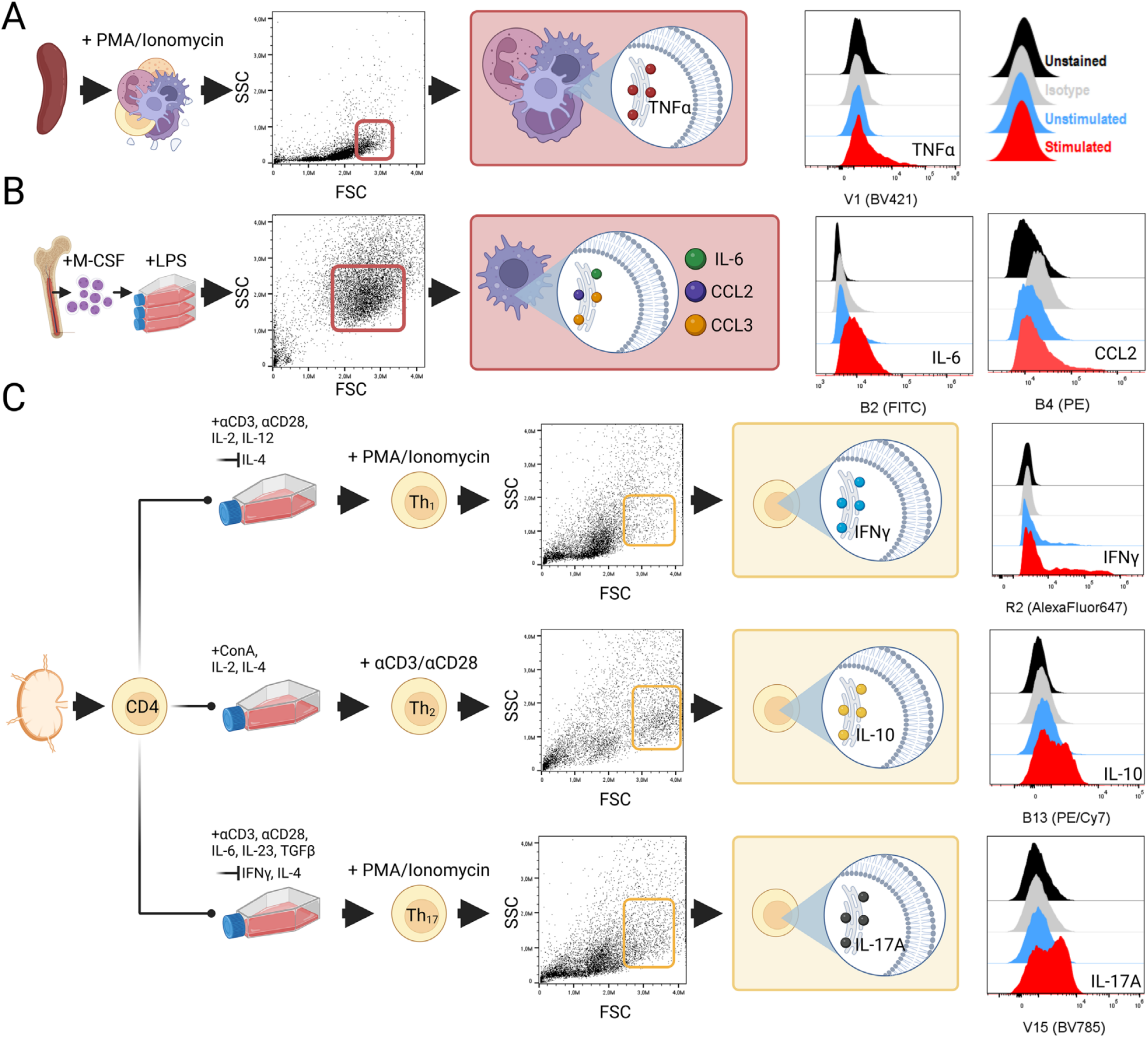
Utilization of in vitro restimulated primary cells for single intracellular cytokine stainings. (A) Splenocytes were stimulated with PMA and ionomycin. FSC^hi^ splenocytes (i.e., myeloid cells) were gated for the assessment of tumor necrosis factor (TNF)α expression. Histograms display the background fluorescence (black) for the indicated channels, along with fluorescence intensities following labeling with isotype controls (grey) and labeling with specific antibodies applied to unstimulated cells (blue) or stimulated cells (red). Cells incubated with isotype controls were stimulated. (B) Bone marrow‐derived macrophages were used for the single labeling of IL‐6, C–C motif chemokine ligand (CCL)2, and CCL3, respectively, subsequent to restimulation using lipopolysaccharide (LPS). (C) Type 1 helper T (Th_1_) cells were obtained following the differentiation of CD4^+^ cells using CD3/CD28‐activating antibodies, IL‐2, IL‐12, and an IL‐4 blocking antibody. Th_1_ cells were restimulated with PMA/ionomycin and subjected to the labeling of interferon (IFN)γ. Th_2_ cells were differentiated by concanavalin A (ConA), IL‐2, and IL‐4 and were subsequently restimulated with CD3/CD28‐activating antibodies. Th_2_ cells were used for the assessment of IL‐10 expression. Th_17_ cells were differentiated using CD3/CD28 activating antibodies, IL‐6, IL‐23, TGFβ, and IFNγ‐ and IL‐4 blocking antibodies. Following restimulation with PMA/ionomycin, Th_17_ cells were used for the evaluation of IL‐17A expression.

To validate the quality of ICS antibody conjugates, in vitro re‐stimulation experiments were performed. Stimulated and unstimulated cells were compared, either unstained, labeled with ICS conjugates, or, in the case of stimulated cells, mixed with the corresponding isotype controls. As shown in Figure 1A, the restimulation of splenocytes with PMA and ionomycin (PI) in the presence of Brefeldin A yielded TNFα^+^ cells that were clearly distinguishable from cells from control experiments. Likewise, LPS restimulation of BMDMs led to the enrichment of cells that expressed IL‐6, CCL2, or CCL3 (Figure 1B and Figure S2B). For the labeling of IFNγ, we first sorted CD4^+^ cells from lymph nodes and subsequently in vitro‐polarized them into type 1 helper T cells (Th_1_, Figure 1C). Compared with controls, an enrichment of IFNγ‐producing cells was induced following PI restimulation. Similarly, Th_2_ and Th_17_ cells were differentiated for the labeling of IL‐10 and IL‐17A, respectively. Restimulation of Th_2_ cells by CD3/CD28 engagement led to robust IL‐10 overexpression (Figure 1C). Similarly, a population of IL‐17A^+^ cells was observed for PI‐restimulated Th_17_ cells (Figure 1C). In order to verify our T‐cell polarization cultures and that Th subtypes predominantly express their cognate cytokines, we conducted cross‐labeling experiments. As shown in Figure S2C, IFNγ labeling was specific for Th_1_ cells, while Th_2_ and Th_17_ cells did not express this cytokine. Likewise, only Th_2_ cells upregulated IL‐10 and only Th_17_ cells upregulated IL‐17A (Figure S2C).

Using the primary cell isolates and cultures specified above, we next tested different concentrations of antibody‐fluorophore conjugates to economize the panel and, more importantly, optimize labeling resolution while reducing spillover spread errors. In detail, we chose for surface labelings a simple approach by using the amounts recommended by the manufacturers as a starting point and reduced them by 50% and 75%, respectively, testing a total of three different conjugate concentrations. We then compared the labeling qualities by assessing staining indices as a measure for the separation and relative distribution of labeled and unlabeled cells (see methods). We suspected that the staining resolution for CD3:BV570 would be considerably reduced following stimulation and thus compared surface labeling (i.e., prior to fixation) to labeling after fixation. As demonstrated in Figure S3A,B, unfavorable resolution of surface CD3:BV570 following stimulation was improved by labeling after fixation and permeabilization. Figure S3C summarizes the labeling results for the remaining surface antigens and depicts the antibody concentrations for all conjugates that yielded favorable staining qualities and were henceforth used for multicolor experiments. For the majority of conjugates, we opted for antibody concentrations that produced the highest staining indices. However, for CD4:BV650, CD11b:BV750, Gr‐1:SparkBlue550, Gr‐1:BV650, CD45:APC/Fire810, and CD31:SuperBright43 bimodal separation was achieved using lower antibody amounts. Although these concentrations resulted in reduced staining indices, they were chosen to economize the panel. For CD90.2:BV711, we selected a concentration with a slightly lower staining index to prevent signal events from nearing the upper limit of the detector's dynamic range.

Subsequently, the above‐employed restimulation cultures were used for testing different ICS antibody conjugate concentrations. We expected that cytokines would be expressed in a low density. We used the recommended concentrations as a baseline and either doubled or halved them accordingly. Staining indices were again used as a proxy for the labeling quality of each tested antibody concentration. Figure S4 summarizes the results and shows the concentrations that were selected for multicolor labeling. For CCL3:APC, a single concentration was assessed, and it was determined that this concentration was capable of producing a convincing bimodal expression.

To conduct multicolor labeling experiments with the panels described above, we chose reference controls for spectral signature deconvolution (i.e., unmixing) based on the outcomes of single labeling experiments. As listed in Table S2, we chose splenocytes, BMDMs, activated T cells, small intestinal isolates, and FLS for most surface antigens. Importantly, all cell‐ and bead‐based reference controls for surface antigens were subjected to fixation and permeabilization. Conversely, the CD3:BV570 conjugate reference control was performed after fixation due to a superior labeling resolution compared with surface staining, as described above. Apart from the TNFα:BV421 reference control, which was generated with PI‐stimulated splenocytes, we chose bead‐based reference controls for most ICS antibodies to obtain the strongest possible signal for optimal unmixing. Taken together, we designed two panels for the study of cytokine expressions among immune and stromal cells. Optimization was performed on a selection of cell isolates and cultures that were expected to express the desired continually expressed and inducible antigens.

### 2.2 Validation of Single‐Cell Cytokine Analysis: Substituting Animal Disease Models with Primary Restimulation Cell Cultures

**FIGURE 2:**
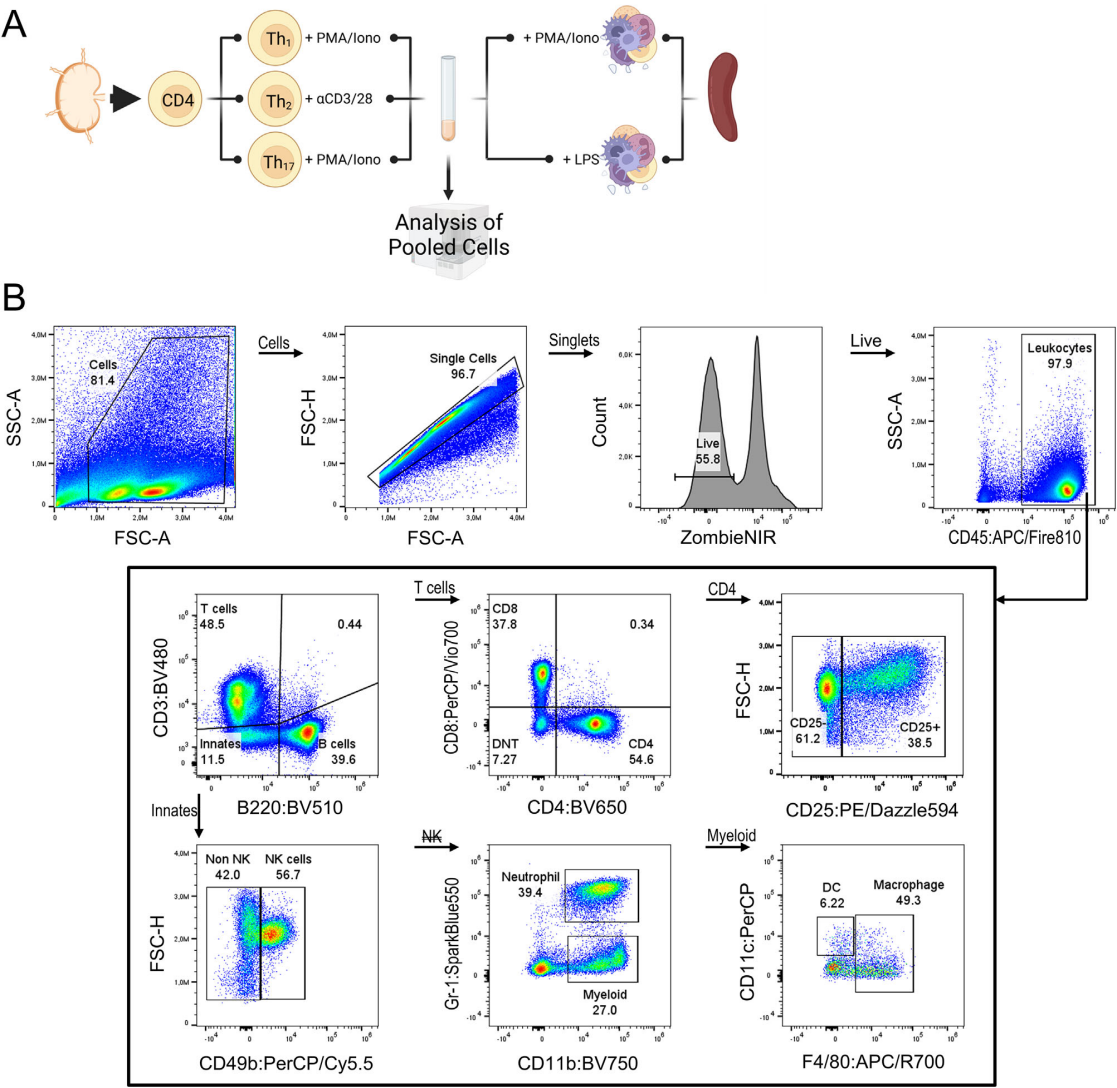
Evaluation of multicolor labeling of surface and intracellular antigens for Panel 1 using simple cell culture models. (A) Experimental scheme. CD4^+^ T cells were purified from lymph nodes and subjected to three differentiation cultures leading to the generation of Th_1_, Th_2_, and Th_17_ cells, respectively. Th_1_ and Th_17_ were subsequently restimulated with PMA and ionomycin. Th_2_ restimulation was achieved by CD3/CD28‐activating antibodies. Freshly isolated splenocytes were restimulated in parallel with PMA/ionomycin or LPS. The separately restimulated cells were then collected in a single tube for multicolor labeling and flow cytometry analysis. (B) Hierarchical gating strategy. Live leukocytes were identified as CD45^+^ singlets. Manually gated subpopulations were denoted as B220^+^ B cells, CD4^+^CD25^−^ T cells, CD4^+^CD25^+^ T cells, CD8^+^ T cells, CD4^−^CD8^−^ double negative T cells (DNT), CD49b^+^ natural killer (NK) cells, CD11b^+^Gr‐1^+^ neutrophils, CD11b^+^CD11c^+^ dendritic cells (DC), and CD11b^+^CD11c^±^F4/80^+^ macrophages.

As quality controls for multicolor surface antigen labeling combined with ICS might be difficult to accomplish by merely using freshly isolated cells from naïve or healthy mice, we chose to combine primary culture models with common restimulation protocols (Figure 2A). This approach not only addresses the challenges of quality control for ICS but also offers an alternative to the commonly employed practice of utilizing animal disease models to procure activated cytokine‐expressing cells. In detail, we polarized CD4^+^ lymph node cells into Th_1_, Th_2_, or Th_17_ cells and subsequently subjected them to restimulation as described above. Freshly isolated splenocytes were restimulated in parallel with either PI or LPS. Following this, polarized helper T cells and splenocytes were pooled and jointly labeled for surface antigens and ICS using Panel 1. We selected this combination of cells to replicate a biological environment similar in complexity to in vivo‐generated samples. We expected that this model would allow us to create a scenario in which all surface and intracellular markers from Panel 1 would be expressed. Figure 2B shows the gating strategy for a representative sample and introduces the immune cell subpopulations that were later analyzed for cytokine expressions. Specifically, we defined CD3^−^B220^+^ B cells, CD3^+^CD4^+^CD25^−^ T cells, CD3^+^CD4^+^CD25^+^ T cells, CD3^+^CD4^−^CD8^−^ double negative T cells (DNTs), CD3^+^CD8^+^ T cells, CD3^−^B220^−^CD49b^+^ NK cells, CD3^−^B220^−^CD11b^+^Gr‐1^+^ neutrophils, CD3^−^B220^−^CD11b^+^CD11c^+/−^F4/80^+^ macrophages and CD3^−^B220^−^CD11b^+^F4/80^−^CD11c^+^ dendritic cells (DCs). In order to get a first impression of our data, we first analyzed global expression values based on median fluorescence intensities (MFI) among all live leukocytes and compared stimulated (STIM group) and unstimulated cells (CON group). We found that restimulation led to the reduction of CD86, Gr‐1, IL‐6, and CCL2 expression (Figure S5).

**FIGURE 3:**
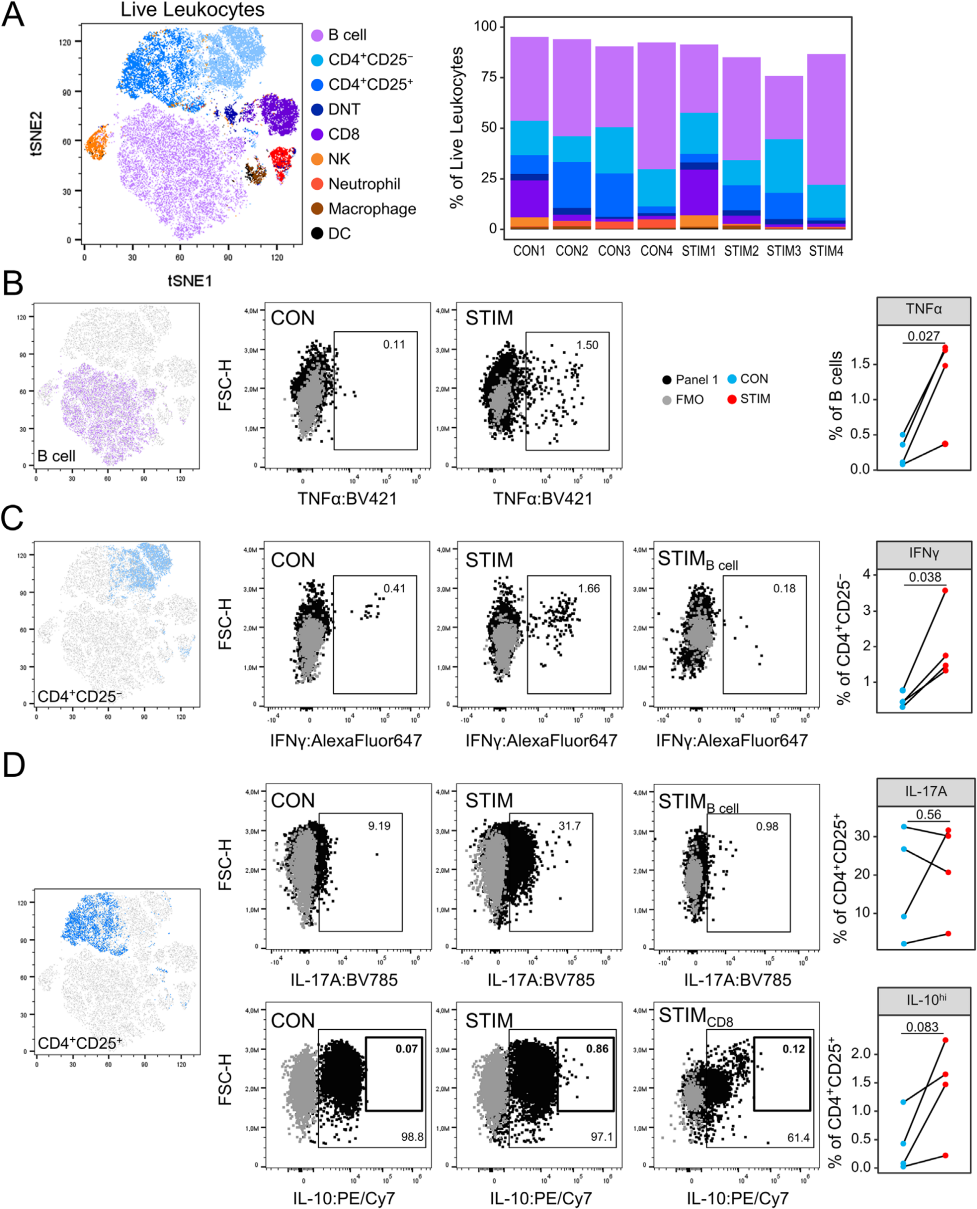
Intracellular cytokine analysis among lymphocyte subpopulations in a culture restimulation model. In vitro‐polarized Th cells and freshly isolated splenocytes were restimulated separately followed by conjoined multicolor labeling and flow cytometry analysis. Data was generated from two independent experiments. (A) Dimensionality reduction by t‐distributed stochastic neighbor embedding illustrates the topological distribution of manually gated leukocyte subpopulations (left panel). The stacked bar plots show the distribution of subpopulations for each sample of the control group (CON) and the restimulation group (STIM), respectively (right panel). (B–D) Representative pseudocolor plots and dot plots show the proportions of cells that expressed TNFα among B cells (B), IFNγ among CD4^+^CD25^−^ T cells (C), and IL‐17A as well as IL‐10 among CD4^+^CD25^+^ T cells (D). Gating boundaries were set using Fluorescence Minus One (FMO) controls. Internal control populations that were expected to have a decreased expression for a given cytokine were included in order to control for unspecific conjugate binding. These were B cells for IFNγ and IL‐17A (C, D) and CD8 T cells for IL‐10 (D). *p*‐values resulted from paired *t*‐tests.

Next, we analyzed the above‐specified immune cell populations in more detail. Of note, by using our gating and immune cell stratification strategy, we were able to cover 88.9% ±6.3% of all live leukocyte events, most of which were B or T lymphocytes (Figure 3A). To establish proper gating boundaries, Fluorescence Minus One (FMO) controls were performed for ICS conjugates. For all cytokines except TNFα—which was expressed by virtually every immune cell subpopulation upon stimulation—we included internal control cells with expected reduced expressions for the other cytokines (e.g., B cells for IL‐17A). This approach enabled us to evaluate nonspecific conjugate binding, which could have been introduced by stimulation‐induced changes in the physical properties of cells. When comparing paired samples before (CON) and after stimulation (STIM), we detected an enrichment of TNFα^+^ B cells (Figure 3B). Among CD4^+^CD25^−^ T cells, we found an increase in IFNγ production upon restimulation that was absent in internal control B cells (Figure 3C). IL‐17A was upregulated in two out of four restimulated samples in CD4^+^CD25^+^ T cells (Figure 3D). B cells again served as an internal control and exhibited no IL‐17A expression. Surprisingly, a high frequency of IL‐10^+^ cells was found for almost every cell population. For instance, the majority of CD4^+^CD25^+^ T cells expressed this cytokine irrespective of stimulation. However, stimulation induced a slight enrichment of a separate IL‐10^hi^ population among CD4^+^CD25^+^ T cells (Figure 3D). CD8^+^ T cells served as an internal control as they had a lower IL‐10^+^ frequency. CD4^+^CD25^+^T cells furthermore upregulated TNFα in the STIM group (Figure S6A). TNFα was also induced in DNTs by restimulation (Figure S6B).

**FIGURE 4:**
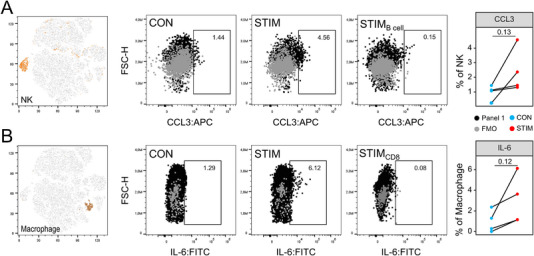
Cytokine expression in innate immune cells from restimulated cell cultures. In vitro‐polarized Th cells and freshly isolated splenocytes were restimulated separately followed by conjoined multicolor labeling and flow cytometry analysis. Data were generated from two independent experiments. (A, B) Representative pseudocolor plots and dot plots show the proportions of cells that expressed CCL3 and IL‐6 among NK cells (A) and macrophages (B), respectively. CON: nonstimulated cultures. STIM: restimulated cultures. Gating boundaries were set using Fluorescence Minus One (FMO) controls. Internal control populations that were expected to have a decreased expression for a given cytokine were included in order to control for unspecific conjugate binding. These were B cells for CCL3 (A) and CD8 T cells for IL‐6 (B). *p*‐values resulted from paired *t*‐tests.

When focusing on innate immune cells, we found a slight enrichment of CCL3^+^ NK cells (Figure 4A). Conversely, B cells did not upregulate this cytokine and were thus selected as internal control cells. For neutrophils, we detected a stimulation‐induced upregulation of TNFα and observed that the majority of neutrophils were IFNγ^+^ irrespective of restimulation (Figure S7A). Restimulation further led to an enrichment of IL‐6^+^ macrophages (Figure 4B). CD8 T cells were used as an internal control since they showed no substantial IL‐6 expression. Macrophages also upregulated TNFα expression upon restimulation, whereas CCL2^+^ macrophages were more frequent in only two out of four samples (Figure S7B). Moreover, restimulation induced an increase in IL‐6^+^ DCs (Figure S7C). Collectively, by using simple restimulation cultures as a strategy to perform multicolor panel validation, we were able to investigate inducible cytokine expression across different immune cell subpopulations without performing stressful animal testing.

### 2.3 Replacing Animal Experimentation for the Validation of Single‐Cell Cytokine Analysis Using Stimulated Co‐cultures of Stromal and Immune Cells

**FIGURE 5:**
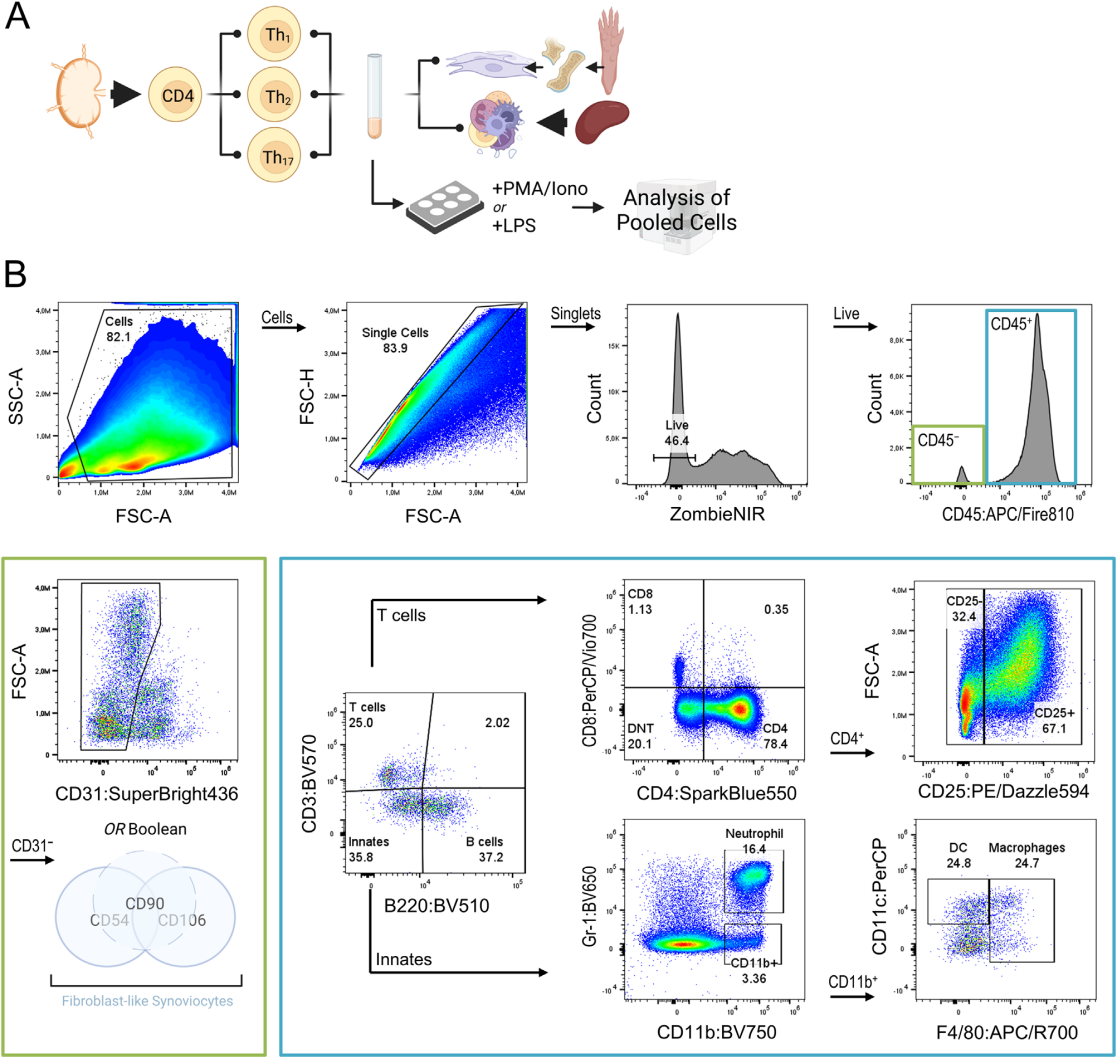
Evaluation of multicolor labeling of surface and intracellular antigens for Panel 2 using a simple co‐culture model. (A) Experimental scheme. CD4^+^ T cells were extracted from lymph nodes and subsequently differentiated into Th_1_, Th_2_, or Th_17_ cells. Helper T cells were then combined with fibroblast‐like synoviocytes and splenocytes followed by joint restimulation with either PMA/ionomycin or LPS. Co‐culture cells were then collected for multicolor labeling and flow cytometry analysis. (B) Hierarchical gating strategy. Fibroblast‐like synoviocytes were identified as CD45^−^CD31^−^ live singlets that expressed any given combination of CD90, CD54, and CD106 as determined by Boolean gating. Live CD45^+^ leukocyte singlets were subdivided into B220^+^ B cells, CD4^+^CD25^−^ T cells, CD4^+^CD25^+^ T cells, CD8^+^ T cells, CD4^−^CD8^−^ DNTs, CD11b^+^Gr‐1^+^ neutrophils, CD11b^+^CD11c^+^ DCs, and CD11b^+^CD11c^−/+^F4/80^+^ macrophages.

In order to simultaneously explore cytokine expressions in FLS and leukocytes using Panel 2, we developed a co‐culture model in which FLS, in vitro‐generated helper T cells, and primary splenocytes were restimulated together either with PI or LPS (Figure 5A). Using this approach, we developed an alternative to the above‐mentioned protocol of separately stimulated cells. This allowed us to validate a comprehensive panel for single‐cell cytokine analysis while avoiding the use of animal disease models. Figure 5B illustrates the gating strategy of the resulting flow cytometry data. The classification of CD45^+^ subpopulations mostly resembled the approach for Panel 1. FLS were defined by Boolean gating as CD45^−^CD31^−^ cells that expressed any given combination of CD90, CD54, and CD106 (Figure S8). Both stimulation regimens (PI and LPS) similarly led to the reduction of global surface and intracellular antigen expressions, namely CD11b, CD86, CD54, IFNγ, IL‐10, and CCL3 (Figure S9).

**FIGURE 6:**
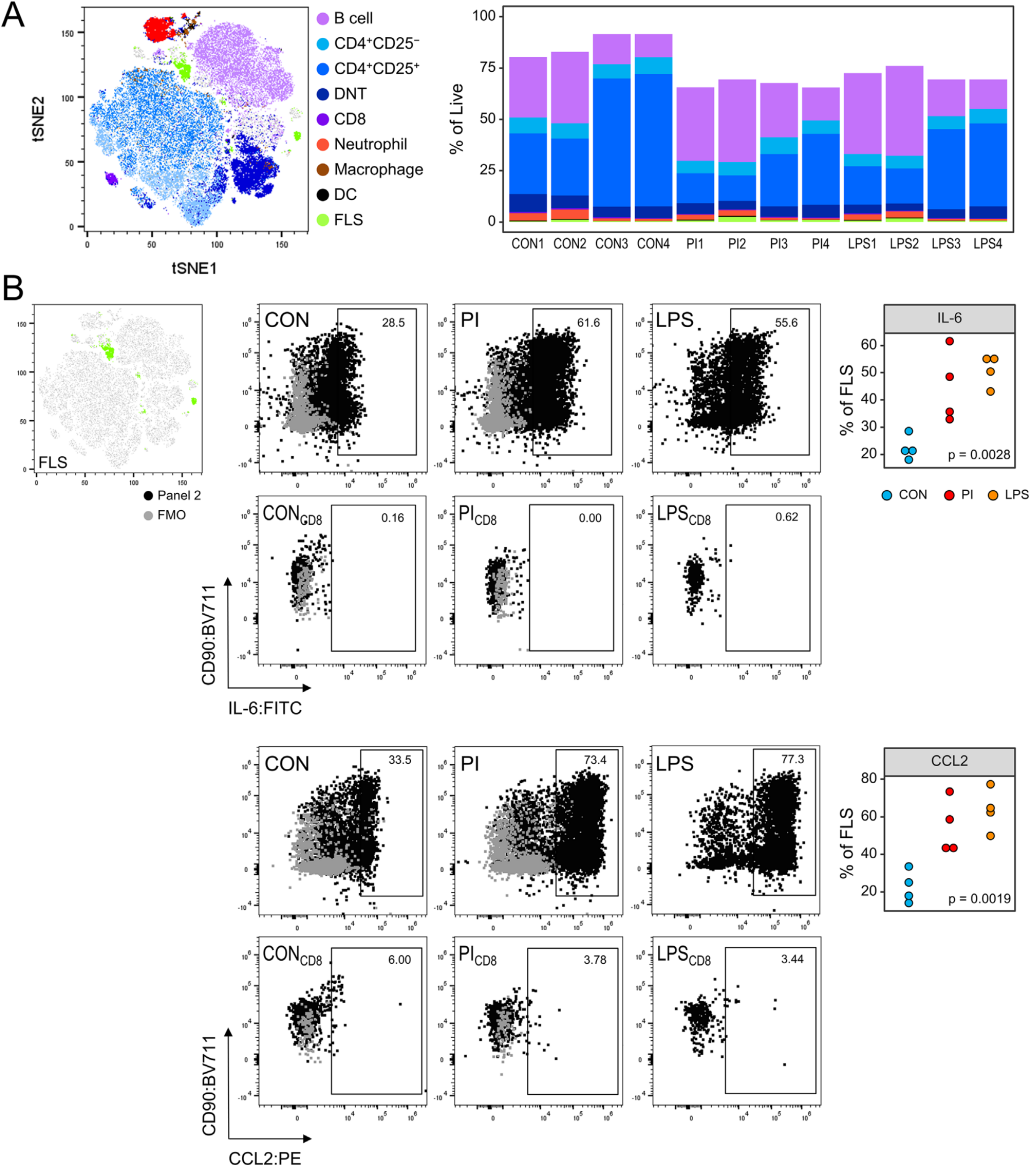
Cytokine expression by Fibroblast‐like synoviocytes upon restimulation. In vitro‐polarized Th cells, fibroblast‐like synocioytes (FLS), and freshly isolated splenocytes were restimulated followed by multicolor labeling and flow cytometry analysis. Data were generated from two independent experiments. (A) Dimensionality reduction by t‐distributed stochastic neighbor embedding illustrates the topological distribution of live single cells that were classified either by manual or Boolean gating (left panel). The stacked bar plot shows the distribution of subpopulations for each sample of the control group (CON), the PMA/ionomycin restimulation group (PI), and the LPS restimulated group, respectively (right panel). (B) Representative pseudocolor plots and dot plots show the proportion of cells that expressed IL‐6 and CCL2 among FLS. Gating boundaries were set using Fluorescence Minus One (FMO) controls for CON and PI‐stimulated cells only. In order to check for unspecific binding of IL‐6 and CCL2 antibodies, CD8 T cells were used as internal control cells. *p*‐values resulted from a one‐way analysis of variance.

Figure 6A depicts the topological distribution of the above‐specified cell populations and further illustrates that the samples were mostly comprised of lymphocytes as expected. Moreover, the results show that 75.2% ±9.4% of all events from live singlets were covered by our cell classification approach. This coverage was lower for restimulated co‐cultures, mostly due to an unexpected reduction in CD4^+^CD25^+^T cell frequencies (Figure 6A). We were curious whether FLS would be activated by either restimulation regimen and detected that both PI and LPS induced the overexpression of IL‐6 and CCL2, respectively (Figure 6B). Gating boundaries were established using FMO controls applied to both CON and PI‐stimulated cells. To account for nonspecific binding of ICS conjugates, CD8 T cells were used as an internal control for IL‐6 and CCL2, revealing negligible signal levels (Figure 6B). We next focused on adaptive immune cells and found that PI and LPS led to the enrichment of TNFα^+^ B cells (Figure S10A). Initiation of TNFα expression among CD4^+^CD25^−^ and CD4^+^CD25^+^T cells was only surveyed following restimulation with PI (Figure S10B,C). IFNγ and IL‐17A overexpression in CD4^+^CD25^+^T cells was induced by both restimulation strategies, although the frequencies of IFNγ^+^ and IL‐17A^+^ were similar between all groups (Figure S10D). When examining cytokine expressions among DNTs, we detected an enrichment of TNFα^+^ cells following PI restimulation, whereas CCL3 overexpression was effectively induced by LPS (Figure S11A,B). Lastly, we analyzed myeloid cells and discovered for neutrophils an enrichment of TNFα^+^ and IL‐6^+^ cells, especially in LPS‐stimulated co‐cultures (Figure S12A). CCL2^+^ neutrophils, however, were more frequent after PI restimulation. LPS was more effective at provoking TNFα expression in both macrophages and DCs, while restimulation effectively prompted CCL2 and CCL3 overexpression in DCs only (Figure S12B–D). In summary, we developed a co‐culture model involving FLS, helper T cells, and splenocytes, stimulated together to explore cytokine expressions. This method provided an alternative to animal disease models for validating a comprehensive panel for single‐cell cytokine analysis.

### 2.4 In Vivo Application of Brefeldin A Was Safe for Mice and Resulted in Only Minor Changes in the Immune and Cytokine Landscapes of Spleen, Blood, and Liver

Injecting Brefeldin A into animals with certain pathologies allows for the study of cytokine expression dynamics in a more physiological context, compared with the conventional restimulation of ex vivo cultured cells. Due to the lack of available safety data concerning the in vivo application of this potentially noxious protein transport inhibitor, we sought to examine adverse events in naїve mice. For this, male C57BL/6J mice were intraperitoneally injected with 10 mg per kg bodyweight Brefeldin A and were subsequently monitored every 30 min for four hours (Figure S13A). Control mice did not receive the injection. We did not detect any disturbances in the appearance and spontaneous behavior of the animals that might indicate distress (data not shown), suggesting that the here‐selected dosage and observation period were safe for mice.

Next, we wondered whether the administration of Brefeldin A impacted surface and intracellular antigen expressions as well as immune cell compositions. We therefore isolated cells from the spleen, peripheral blood, and liver and labeled them for flow cytometry analysis using Panel 1 and the above‐described gating strategy. Figure S13B shows for all samples a principal component analysis of global MFI values among live leukocytes and illustrates that differences between samples were mainly due to tissue type rather than administration of Brefeldin A. A more detailed analysis of the data revealed that, apart from a moderate increase in liver DCs in Brefeldin A‐treated mice (Figure S13C), administration of the drug had no significant effect on the abundance of immune cell populations in the organs studied. Cytokine expression values were also mostly unaltered. However, compared with untreated mice, we detected decreased MFIs of CCL2 in B cells, DNTs, and neutrophils as well as a decreased MFI of CCL3 in blood DCs (Figure S13D). Additionally, IL‐10^+^ macrophages and CCL2^+^ neutrophils were less abundant in blood samples from Brefeldin A‐treated mice (Figure S13E). Application of the agent further induced a minor increase of IL‐6^+^ NK cells from the liver. We here demonstrated that Brefeldin A administration was safe and had no substantial impact on immune cell frequencies or cytokine expression in the spleen, peripheral blood, and liver, making it well‐suited for in situ cytokine analysis.

## 3 Discussion

Examining cytokines at the single‐cell level provides unique insights into the functional characteristics of immune and stromal cells, as well as their coordination during physiological and aberrant immune responses [19]. In this study, we developed two panels to investigate mouse cytokine expressions across adaptive and innate immune cell populations, as well as joint cells. To ensure optimal fluorophore‐to‐marker allocations, we adhered to panel design guidelines detailed elsewhere [4, 20, 21]. We carefully designed the panels to ensure that the combination of fluorophores resulted in a low complexity index with minimal spillover spread [4], thereby increasing the likelihood of obtaining optimal labeling results. Our particular emphasis was on achieving satisfactory resolutions for intracellularly trapped cytokines. Notably, Panel 1 was designed for general immunophenotyping, while Panel 2 represents a more specialized assay that incorporates the study of joint cells. Both panels, however, include the labeling of cytokines that orchestrate a wide range of biological functions across various physiological and pathophysiological scenarios. Depending on the research question, we suggest incorporating additional markers into the here reported panels. For instance, adding CD127 for detectors V3 or B7 would enable the analysis of innate lymphoid cells, which have recently emerged as a focus of novel immunobiology research and can be classified according to their cytokine expression patterns [22].

Despite often acting in concert, even at very low concentrations, various cytokines might not be produced with identical kinetics in response to an immunological insult, posing a challenge to their simultaneous detection [23, 24, 25]. Hence, the application of compounds that inhibit intracellular protein transport is required for the accumulation of cytokines, thus enabling their synchronized visualization by fluorophore‐labeled antibodies and flow cytometry. Moreover, these compounds facilitate the detection of cytokines by enriching their concentration within the cell [26]. Single‐cell cytokine analysis therefore relies on the use of Monensin or Brefeldin A. The utilization of these compounds in combination with cytokine‐inducing stimulation is almost exclusively restricted to ex vivo methodology [8, 27, 28]. Accordingly, only a limited number of studies incorporated the injection of comparably less toxic Brefeldin A into live animals [9, 14, 15, 17], although this approach holds the potential to further our understating of in situ cytokinogenesis. Surprisingly, we found no data regarding the impact of short‐term Brefeldin A incubation on the immune homeostasis of healthy mice, nor did we find any information on its effects on animal wellbeing. We have demonstrated that mice exposed to this agent for not more than four hours showed no signs of distress. Brefeldin A further introduced only minor changes into the basic immune landscape. It should be noted that we did not aim to provide evidence for the feasibility of Brefeldin A injection into live animals for single‐cell cytokine analysis, as this has already been repeatedly demonstrated [9, 14, 15, 17]. Instead, our primary objectives were twofold: first, to establish a foundation for application to committees for animal research and ethics by demonstrating that Brefeldin A does not cause distress in mice, and second, to illustrate the magnitude of bias introduced by this compound into the immune landscapes of mice. Importantly, the panels presented here were specifically designed for use on samples obtained from Brefeldin A‐treated mice, underscoring the necessity of establishing safety data in the context of our work. The incorporation of in vivo Brefeldin A application enables the analysis of in situ cytokine production while preserving tissue integrity and the interactions between stromal and immune cells. This approach allows for the study of cellular effector functions in a more physiologically relevant context compared with conventional ex vivo stimulation [9]. Moreover, this method addresses the limitations of ex vivo methodologies, such as difficulties in achieving simultaneous cytokine induction and tracking. It also circumvents artificially induced alterations in cell morphology caused by stimulants [27, 29, 30].

While eliciting cytokine expression is relatively straightforward in cells from human tissues, as they are continually exposed to microbial stimuli, stimulated immune cells from laboratory animals typically exhibit lower levels of cytokine production [31]. This disparity arises from the pathogen‐free environment in which animals are usually housed, resulting in a decreased presence of effector cells [32]. As a result, research groups often turn to animal disease models to obtain cells suitable for validating ICS panels [11, 12]. This may raise ethical concerns regarding animal welfare, as the establishment and optimization of panels alone may not yield novel insights into immunobiology, thus potentially leading to an unnecessary increase in the number of animals used. Moreover, validation can be achieved through methods that do not require in vivo experimentation [33]. Our primary aim was to provide researchers with a readily accessible framework to optimize and validate comprehensive panels for multiparametric single‐cell cytokine analysis. Additionally, we described in detail how to circumvent the need for stressful animal testing by exclusively using in vitro stimulation procedures.

In detail, we initially selected suitable primary cell isolates and culture models that would enable the identification of the desired surface and intracellular antigens. For example, common antigens such as CD3 and CD45 were labeled on freshly isolated splenocytes while cytokines were stained following stimulated T cell or macrophage cultures. These samples were subsequently used for testing different concentrations of antibody conjugates. This process serves panel economization and, more importantly, helps determine labeling concentrations that yield optimal resolutions and reduces nonspecific binding, thereby resulting in minimal background signals [34, 35]. This step can often be time‐consuming and requires significant material resources, as it typically involves testing at least four different antibody concentrations [7, 36]. To find a balance between labeling optimization and resource efficiency, we here chose to test three different conjugate amounts based on the manufacturer's instructions. Nonetheless, we would like to underscore the fact that the titration of antibody conjugate concentrations for a given antigen is markedly superior to our approach. In order to maximize signal‐to‐noise ratios and optimize nonspecific binding, it is necessary to test a greater number of concentrations in order to achieve quantitative saturation of antigen binding sites.

The here used primary and cultured cells were further utilized as reference controls for spectral unmixing. In this context, variations in autofluorescence across different cell types highlight the necessity of including matching unstained samples for cell‐based single‐labeling controls. This ensures accurate calculation of spillover corrections, particularly when the sample type in subsequent multicolor labeling experiments differs from the reference control types (e.g., liver cells vs. polarized helper T cells). In case cells only exhibit rare positive events for a given marker, we chose compensation beads as reference controls [4, 37]. Crucially, when creating single‐labeling controls for antibody titrations or unmixing, it is essential to fixate and permeabilize surface‐labeled cell samples or bead surrogates exactly like the final multicolor sample, as these procedures can modify cellular autofluorescence patterns as well as the overall spectral signature of fluorophores [7, 21, 38]. Multicolor validation for both panels was performed using cultures that contained all cell types that were expected to express the desired cytokines. We demonstrated the enrichment of innately and adaptively expressed cytokines among diverse cell subtypes in samples from stimulated cultures. Together, we present a streamlined method for the establishment of two multicolor panels that are designed to analyze cellular cytokine expressions across various mouse organs. These panels comprehensively address a wide spectrum of cell populations, including joint cells, making them versatile tools for studying immunodynamics in infection and autoinflammation, such as sepsis and autoimmune arthritis, respectively [39, 40].

While the multicolor labeling experiments conducted in this study on stimulated co‐cultures produced satisfactory results, some limitations must be acknowledged. To address nonspecific binding—either by the Fc domain or the attached fluorochrome—stimulated cells labeled with ICS conjugates were compared with stimulated cells incubated with corresponding isotype controls [21]. However, the validity of isotype control antibodies as specificity controls has been contested on the grounds that they are unable to account for epitope sharing, which can result in unwanted specific antibody binding and false‐positive signals. [41]. In cases where such issues are likely, researchers may consider using knockout cell lines or isolates from knockout mice. In our study, we opted against this approach for two reasons: (1) cell lines are challenging to compare with primary cells, and (2) sacrificing additional mice for stricter controls conflicted with the 3R principles central to our work. Instead, we directly implemented multicolor experiments. These involved comparing cytokine‐expressing cells after stimulation with other cell types in the same samples that were not expected to express the same cytokine. This strategy helped control nonspecific binding, which might have been amplified by stimulation‐induced changes in physical cell properties [29]. Nevertheless, for a more comprehensive validation of the utilized conjugates, particularly in the context of analyzing antigens that exhibit low levels of expression, it is recommended to employ blocking controls. This can be achieved by introducing an excess of soluble antigen (e.g., recombinant cytokine) or an unconjugated isotype to the samples prior to labeling with the conjugate.

We further observed for both panels a clearly reduced resolution of surface CD3 labeling following stimulation, which seems to be a common phenomenon in single‐cell analysis, particularly affecting T cells [42, 43]. Similar to other studies, we addressed this issue by intracellular labeling of CD3 [23]. Moreover, we were able to phenotypically discriminate only three out of every four cell events in the multilabeling experiment during the validation of Panel 2 (immune cells & FLS). A recent study has shown that an increase in the production of reactive oxygen species by neutrophils, following PMA stimulation, leads to the elimination of co‐cultured T cells [27]. This phenomenon may have influenced the reduction of identifiable lymphocyte populations in our model. This aligns with the validation process for Panel 1, for which we stimulated polarized T‐cell cultures and neutrophil‐containing samples separately and yielded a higher event coverage.

Collectively, we offer researchers from diverse backgrounds an accessible method for multiparametric full spectrum flow cytometry panel validation, which includes intracellular labeling and importantly, helps replace stressful animal testing. Integrating these panels with the in vivo application of Brefeldin A not only promises to reduce animal use by maximizing insights from limited sample material but also unlocks new avenues for investigating cytokine expression dynamics.

## 4 Materials & Methods

### 4.1 Antibodies for Flow Cytometry

(Anti‐mouse)TNFα:BV421 (clone MP6‐XT22), B220:BV510 (RA3‐6B2), CD3:BV570 (17A2), CD80:BV605 (16‐10A1), CD4:BV650 (GK1.5), Gr‐1:BV650 (RB6‐8C5), CD90.2:BV711 (30‐H12), CD11b:BV750 (M1/70), IL‐17A:BV785 (TC11‐18H10.1), Gr‐1:SparkBlue550 (RB6‐8C5), CD4:SparkBlue550 (GK1.5), CCL2:PE (2H5), CD25:PE/Dazzle594 (3C7), CD11c:PerCP (N418), CD49b:PerCP/Cy5.5 (HMα2), CD54:PerCP/Cy5.5 (YN1/1.7.4), IL‐10:PE/Cy7 (JES5‐16E3), IFNγ:AlexaFluor647 (XMG1.2) and CD45:APC/Fire810 (30‐F11) were purchased from Biolegend. CD31:Superbright436 (62‐0311‐80) was obtained from ThermoFisher. CD3:BV480 (17A2), CD106:BV480 (429) and F4/80:APC/R700 (T45‐2342) were purchased from BD Biosciences. IL‐6:FITC (REA1034), CD8:PerCP/Vio700 (REA601), CCL3:APC (REA355) and CD86:APC/Vio770 (PO3.3) were bought from Miltenyi Biotec. The listed antibodies were used for Panel 1 and Panel 2 (Table S1). Isotype control antibodies for the quality assurance of intracellular cytokine staining were Rat IgG1:BV421 (clone RTK2071), Rat IgG1:BV785, Armenian Hamster IgG:PE (HTK888), Rat IgG2b:PE/Cy7 (RTK4530) and Rat IgG1:AlexaFluor647 and were obtained from BioLegend. Human IgG1:FITC (REA293) and Human IgG1:APC were purchased from Miltenyi Biotec. For all flow cytometry analyses as well as panel design and optimization, we here adhered to the “Guidelines for the use of flow cytometry and cell sorting in immunological studies” [21].

### 4.2 Mouse Tissue Isolation

NMRI mice were bred in the animal care facility of the Rudolf‐Zenker‐Institute (Rostock University Medical Center) and were housed under specific pathogen‐free conditions. Animals were kept on a 12 h light/dark cycle and were given food and water ad libitum. Male and female mice that were a maximum of 18 months old were sacrificed by ketamine/xylazine overdose and exsanguination by cardiac puncture under full anesthesia. All NMRI mice used for this study were not subjected to any other procedure that might cause pain, suffering, or distress (see Directive 2010/63/EU). Sacrifices of NMRI mice for tissue isolation were therefore in accordance with Section 4, Paragraph 3 TierSchG (Germany). The fur of the mice was rinsed with 70% ethanol, after which the inguinal, popliteal, axillary, brachial, and cervical lymph nodes (LNs) were removed. Subsequently, the hind‐ and forelimbs were removed by dislocation. Next, the peritoneum was opened and the mesenteric LNs were extracted. After this, the small intestine was truncated below the pylorus and above the caecum. Lastly, the spleen was removed. All organs were transferred and stored in a cell culture medium (DMEM or RPMI, PAN Biotech) on ice.

### 4.3 Activated and Helper T Cell Culture

Spleens and LNs were separated through a 70 µm strainer. Splenocytes were then subjected to erythrocyte lysis according to the manufacturer's instructions (RBC Lysis Buffer from Biolegend). Splenocytes and LN cells were suspended in autoMACS Rinsing Solution (Miltenyi) supplemented with 0.5% BSA. CD4^+^ lymphocytes were isolated by L3T4 MicroBeads using the autoMACS (“posselds”) following the manufacturer's instructions (Miltenyi). All CD4^+^ lymphocyte cultures were performed in RPMI medium with 3 mM stable L‐glutamine and 2 g/L NaHCO_3_ supplemented with 10% FCS (PAN Biotech), 100 U/mL Penicillin, 100 µg/mL Streptomycin (ThermoFisher) and 50 µM β‐Mercaptoethanol (Sigma). Activated T cells were generated by the culture of 0.25 × 10^6^ CD4^+^ lymphocytes per cm^2^ on cell culture plates that were coated with 3 µg/mL anti‐CD3 (clone 145‐2C11, Biolegend) for 2 h at 37°C in Dulbecco's phosphate‐buffered saline (DPBS, ThermoFisher). Additionally, cells were provided with 3 µg/mL anti‐CD28 (clone 37.51), 5 ng/mL human TGFβ1 (Biolegend), and 5 ng/mL IL‐2 (Miltenyi) in 1 mL medium per 10^6^ cells. After 2 days, the same amount of medium containing double the above‐listed concentrations of anti‐CD28, TGFβ1, and IL‐2 was added to the culture.

To facilitate the differentiation and polarization of helper T cells, we adhered to the protocols available from a recombinant cytokine manufacturer (Biolegend; see https://www.biolegend.com/en‐us/protocols/th2‐polarization‐of‐mouse‐cd4‐cells‐protocol). In detail, CD4^+^ lymphocytes were cultured at a density of 10^6^ cells per cm^2^ per 1 mL medium. Culture plates were precoated with 3 µg/mL anti‐CD3 for Th_1_ and 5 µg/mL anti‐CD3 for Th_17_. Th_1_ cells were generated by the addition of 3 µg/mL anti‐CD28, 10 µg/mL anti‐IL‐4 (clone 11B11), 10 ng/mL IL‐12p70 (Biolegend) and 5 ng/mL IL‐2. After a culture of 2 days, the same volume of medium was added without stimulants. For Th_2_ cells, the culture medium was supplemented with 5 µg/mL concanavalin A (ConA, Sigma), 20 ng/mL IL‐2, and 50 ng/mL IL‐4 (Miltenyi). Two days later, cells were washed with DPBS and the culture was continued by adding the stimulants without concanavalin A at the same concentrations. Th_17_ cells were differentiated by adding 3 µg/mL anti‐CD28, 10 µg/mL anti‐IFNγ (clone XMG1.2), 10 µg/mL anti‐IL‐4, 50 ng/mL IL‐6, 5 ng/mL IL‐23 (Miltenyi) and 1 ng/mL TGFβ1 for two days. Subsequently, an equivalent volume of medium containing the same amounts of cytokines and antibodies was added. Cultures of activated or helper T cells all lasted a total of four or 5 days.

### 4.4 Small Intestine Digestion

Small intestines were processed based on a previously described protocol [44]. In brief, the small intestines were flushed with 10–20 mL ice‐cold Hank's Balanced Salt Solution (HBSS). Using scissors, small intestines were opened longitudinally and cut into 2 × 2 mm pieces. The organ fragments were then incubated twice in HBSS (without Ca^2+^/Mg^2+^) containing 1.5 mM DTT (Sigma), 5% FCS, and 5 mM EDTA in a water bath at 37°C for 20 min with vigorous shaking every 5 min. Subsequently, the buffer was removed using a 70 µm strainer and the retained tissue was then incubated in HBSS without supplements for 20 min at 37°C. Following buffer removal, the tissue was incubated for 30–60 min at 400 rpm and 37°C in 5 mL DMEM containing 1 mM Pyruvate, 125 U/mL collagenase type I (ThermoFisher), 400 U/mL collagenase type IV (StemCell), 0.25 U/mL dispase (Sigma) and 15 U/mL DNase I (Roche). The enzymatic reaction was quenched by the addition of autoMACS running buffer (RB, Miltenyi). Small intestine fragments were subsequently passed through a 70 µm strainer and washed with RB.

### 4.5 Fibroblast‐Like Synoviocyte and Bone Marrow‐derived Macrophage Culture

FLS were extracted from paws as previously described [39]. In brief, claws were cut off, the skin was removed and tendons were transected. Before removing the soft tissue, the distal phalanges were detached. Paws were then dislocated from long bones and digested for 60–90 min at 37°C and 350–400 rpm in 2 mL supplemented DMEM (10% FCS, Penicillin/Streptomycin, 2 mM L‐Glutamine, 10 mM HEPES, 1 mM Pyruvate, and 4.5 g/L D‐glucose) containing 240 U/mL collagenase type IV. Bone debris was removed by filtration through a 70 µm strainer. Subsequently, cells were washed, suspended in supplemented DMEM, and seeded at a density of 10,000–20,000 cells per cm^2^. FLS were cultured for at least five days until full confluency. For labeling experiments, cells were washed with DPBS and detached by 0.25% Trypsin (ThermoFisher).

Soft tissue and epiphyses of the femora and tibiae were removed and bone marrow was isolated by centrifugation at 10,000×*g* for 15 s [45]. Bone marrow cells were then seeded into culture plates at a density of 0.3 × 10^6^ cells per cm^2^ in supplemented DMEM. Macrophages were differentiated by the addition of 20 ng/mL M‐CSF (R&D Systems). The stimulus was maintained by medium replacement and M‐CSF supplementation 1 and 4 days later. Cells were harvested after 3 more days using a gentle detachment protocol as previously described [46].

### 4.6 Single Labeling and Testing Different Antibody Concentrations

For the labeling of dead cells, isolated splenocytes were washed with DPBS and incubated for 5 min at 65°C. Subsequently, cells were centrifuged and suspended in 4000‐fold diluted ZombieNIR (Biolegend), followed by incubation at room temperature (RT) for 20 min. Splenocytes were then washed with RB, suspended in 0.5 mL Fixation Buffer (Biolegend), and incubated for 20 min at RT. After centrifugation, cells were washed three times with 0.5 mL intracellular staining permeabilization wash buffer (Biolegend). Cells were analyzed in RB on the 3L Cytek Aurora flow cytometer (Cytek Biosciences).

The antibody amounts listed below refer to the labeling of one million cells in 100 µL. All cells used for surface antigen labeling were first incubated with 10% FCS, 1 µg anti‐CD16/32 (clone 93), and 5% tandem dye‐blocking solution (True Monocyte Blocker, Biolegend) for 15 min on ice. Splenocytes were labeled with CD45:APC/Fire810 (0.25/0.12/0.06 µg), CD3:BV480 (0.5/0.25/0.12 µg), CD3:BV570 (0.5/0.25/0.12 µg), CD4:BV650 (0.25/0.12/0.06 µg), CD4:SparkBlue550 (0.5/0.25/0.125 µg), CD8:PerCP/Vio700 (0.3/0.15/0.075 µg), B220:BV510 (0.5/0.25/0.12 µg), CD49b:PerCP/Cy5.5 (0.06/0.03/0.02 µg), CD11b:BV750 (0.25/0.12/0.06 µg), CD11c:PerCP (0.25/0.12/0.06 µg), CD86:APC/Vio770 (0.3/0.15/0.075 µg), Gr‐1:SparkBlue550 (0.5/0.25/0.125 µg) or Gr‐1:BV650 (0.25, 0.12, 0.06 µg). Activated T cells (see “Activated and helper T cell culture”) were incubated with CD25:PE/Dazzle594 (1/0.5/0.25 µg). BMDMs were labeled with F4/80:APC/R700 (0.5/0.24/0.12 µg) or CD80:BV605 (0.25/0.12/0.06 µg). Small intestinal isolates were used for the labeling with CD31:SuperBright436 (0.25/0.12/0.06 µg). CD90:BV711 (0.06/0.03/0.02 µg), CD54:PerCP/Cy5.5 (0.25/0.125/0.06 µg) and CD106:BV480 (0.5/0.25/0.12 µg) were used for FLS. Incubations with antibodies were performed on ice for 20 min. Cells were subsequently washed with RB, fixed in aqueous formaldehyde, washed with permeabilization buffer, and analyzed as described above. For the purpose of comparing “extracellular” labeling of CD3:BV570 with “intracellular” labeling, some samples were incubated with this conjugate following fixation and permeabilization (see below).

In order to trigger cytokine overexpression, splenocytes were stimulated with 50 ng/mL phorbol‐12‐myristate‐13‐acetate (PMA) and 1 µg/mL ionomycin (ThermoFisher) in the presence of 5 µg/mL Brefeldin A (Biolegend) for four hours at 37°C. The stimulation period and the Brefeldin A concentrations were equivalent for all tested cell types. For BMDMs, 1 µg/mL LPS (Sigma) was added. For Th cells restimulation we adhered to protocols available from Biolegend. Th_1_ cells were stimulated with 50 ng/mL PMA and 1 µg/mL ionomycin (Sigma). Th_2_ were seeded onto culture plates precoated with 10 µg/mL anti‐CD3 and were provided with 5 µg/mL anti‐CD28. Th_17_ cells were stimulated with 500 ng/mL PMA and 500 ng/mL ionomycin. As controls, cultures of Brefeldin A‐treated cells that were not stimulated were performed in parallel. For ICS as well as labeling of CD3:BV570, cells were first formalin‐fixed and permeabilized. Cells were then suspended in a residual volume of permeabilization wash buffer left after decantation. Unspecific binding of antibodies or tandem dyes was blocked as described above. Splenocytes were labeled with TNFα:BV421 (0.25/0.12/0.06 µg), RtIgG:BV421 (0.25 µg) or CD3:BV570 (0.5/0.25/0.12 µg). For BMDMs, IL‐6:FITC (0.3/0.15 µg), HuIgG:FITC (0.15 µg), CCL2:PE (0.5/0.24 µg), HmIgG:PE (0.5 µg), CCL3:APC (0.15 µg) or HuIgG:APC (0.15 µg) was used. Th_1_ cells were labeled with IFNγ:AlexaFluor647 (0.5/0.25/0.12 µg) or RtIgG:AlexaFluor647 (0.25 µg). Th_2_ cells were stained with IL‐10:PE/Cy7 (0.5/0.25/0.12 µg) or RtIgG:PE/Cy7 (0.25 µg). Th_17_ were labeled with IL‐17A:BV785 (0.5/0.25/0.12 µg) or RtIgG:BV785 (0.25 µg). Incubation with ICS antibodies lasted 20 min at RT. Subsequently, cells were washed in a permeabilization buffer, followed by suspension in RB and flow cytometry analysis. Labeling quality was assessed by calculating staining indices (SI) using the following equation:

$$\mathrm{SI}\;=\frac{\left({\mathrm{MFI}}_{\mathrm{pos}}-{\mathrm{MFI}}_{\mathrm{neg}}\right)}{2\times \sigma_{\mathrm{neg}}},$$

where MFI_pos_ and MFI_neg_ are the median fluorescence intensities of positively and negatively labeled cells, respectively. The standard deviation of the negative population is denoted with *σ*_neg_.

To procure bead‐based reference controls, one drop of UltraComp eBeads (Thermo) was mixed with 1 µL of hybridoma‐based antibody conjugates (IL‐17A:BV785, CCL2:PE, IL‐10:PE/Cy7, IFNγ:AlexaFluor647). The mixture was incubated on ice in the dark for 20 min, followed by washing with permeabilization buffer, resuspension in RB, and analysis via flow cytometry. For REA‐based conjugates (IL‐6:FITC, CCL3:APC), the MACS Comp Bead Kit, anti‐REA (Miltenyi) was used following the manufacturer's instructions.

### 4.7 Restimulation Cultures and Multicolor Labeling

For each restimulation regimen, 10^6^ cells were used. CD4^+^ cells were extracted from lymph nodes, differentiated into Th_1_, Th_2,_ and Th_17_ cells, and restimulated as described above. Splenocytes were restimulated in parallel either with 20 ng/mL PMA and 1 µg/mL ionomycin or with 1 µg/mL LPS. All cell types were additionally provided with 5 µg/mL Brefeldin A. After 4 of incubation, all cells from each culture were pooled into a single tube. From this pool, 10^6^ cells were used for multicolor labeling. Cells were washed in DPBS and dead cells were labeled using ZombieNIR. After washing with RB, unspecific conjugate binding was blocked. Subsequently, cells were incubated with 0.5 µg CD3:BV480, 0.5 µg B220:BV510, 0.25 µg CD80:BV605, 0.06 µg CD4:BV650, 0.06 µg CD11b:BV750, 0.125 µg Gr‐1:SparkBlue550, 1 µg CD25:PE/Dazzle594, 0.25 µg CD11c:PerCP, 0.03 µg CD49b:PerCP/Cy5.5, 0.3 µg CD8:PerCP/Vio700, 0.12 µg F4/80:APC/R700, 0.3 µg CD86:APC/Vio770, and 0.06 µg CD45:APC/Fire810 for 20 min on ice. Following this, cells were fixed, permeabilized, and unspecific conjugate binding was blocked. ICS was performed by incubation with 0.25 µg TNFα:BV421, 0.5 µg IL‐17A:BV785, 0.3 µg IL‐6:FITC, 0.5 µg CCL2:PE, 0.5 µg IL‐10:PE/Cy7, 0.15 µg CCL3:APC and 0.5 µg IFNγ:AlexaFluor647 for 20 min at RT. In some samples, 0.5 µg CD3:BV570 was applied together with ICS antibodies (Panel 2, only). Cells were subsequently analyzed by flow cytometry.

In order to perform stimulation of co‐cultures, FLS, Th_1,_ and Th_17_ cells were obtained as described above. Th_2_ cells were differentiated from CD4^+^ lymph node cells by culture on anti‐CD3 coated plates (10 µg/mL) and by the addition of 3 µg/mL anti‐CD28, 20 ng/mL IL‐2 as well as 50 ng/mL IL‐4 for 2 days. Cells were then washed and cultured for another 2 to 3 days in the presence of the same concentrations of IL‐2 and IL‐4. FLS were detached by trypsinization and 0.3 × 10^6^ cells were seeded in each well of a six‐well culture plate followed by incubation overnight. On the following day, FLS were combined with 2 × 10^6^ pooled lymphocytes consisting of equal numbers of Th_1_, Th_2_, Th_17_ cells, and splenocytes (0.5 × 10^6^ of each cell type). Co‐cultures were stimulated for 4 in the presence of 5 µg/mL Brefeldin A with either 50 ng/mL PMA and 500 ng/mL ionomycin or with 1 µg/mL LPS. After obtaining lymphocytes and detaching FLS using trypsin, cells were again combined, washed with DPBS, and dead cells were labeled with ZombieNIR. Blocked cells were then incubated with 0.12 µg CD31:SuperBright436, 0.5 µg CD106:BV480, 0.5 µg B220:BV510, 0.5 µg CD3:BV570, 0.25 µg CD80:BV605, 0.06 µg Gr‐1:BV650, 0.03 µg CD90:BV711, 0.06 µg CD11b:BV750, 0.125 µg CD4:SparkBlue550, 1 µg CD25:PE/Dazzle594, 0.25 µg CD11c:PerCP, 0.125 µg CD54:PerCP/Cy5.5, 0.3 µg CD8:PerCP/Vio700, 0.12 µg F4/80:APC/R700, 0.3 µg CD86:APC/Vio770 and 0.06 µg CD45:APC/Fire810 for 20 min on ice. Fixation, permeabilization, ICS, and CD3 labeling were performed as described above.

### 4.8 In Vivo Application of Brefeldin A and Organ Excision

Brefeldin A (Sigma) was solved in DMSO (Merck) to a concentration of 20 mg/mL. The solution was then diluted in sterile DPBS to a final concentration of 1 mg/mL Brefeldin A and 5% DMSO. Six to 8‐week‐old male C57BL/6J mice were intraperitoneally administered 10 mg per kg body weight of Brefeldin A (*n* = 5), which corresponded to an injection volume of no more than 10 mL per kg body weight. Mice were subsequently monitored for adverse effects every 30 min for 4 h, with behavior and appearance serving as indicators of overall health and distress level. Animal experiments were reviewed and approved by the federal state's animal ethics committee (State Department of Agriculture, Food Safety and Fishery in Mecklenburg‐Western Pomerania) under the file reference number 7221.3‐1‐042/22. Age‐ and sex‐matched control mice (no substance application, *n* = 3) were sacrificed for tissue isolation in accordance with Section 4, Paragraph 3 TierSchG (Germany) as stated above. Blood was drawn by cardiac puncture, mixed with 10% EDTA, and stored on ice. The spleen and liver were extracted and stored in RB on ice. Spleens were processed as stated above. Blood (200 µL) was subjected to erythrocyte lysis by the addition of 5 mL ice‐cold lysis buffer (150 mM NH_4_Cl, 10 mM NaHCO_3_, and 1 mM EDTA) and incubation at room temperature for 10 min under gentle agitation followed by centrifugation and suspension in RB. Liver samples were cut into small pieces using a scalpel and 0.4 g of the organ was digested in 2 mL of 1 mg/mL Collagenase/Dispase (Roche) for 30 min at 37°C and 400 rpm. Subsequently, organ fragments were separated through a 100 µm strainer. Liver cells were centrifuged and erythrocyte lysis was performed in the same fashion as for splenocytes. Single‐cell suspensions from all organs were counted and labeled for surface and intracellular antigens using the antibody‐fluorophore conjugate concentrations that yielded satisfactory staining results from Panel 1 as described above.

### 4.9 Statistical Analysis

Flow cytometry data were analyzed and visualized in FlowJo (v10.8.1). Dimensionality reduction was performed using the DownSampleV3 and t‐distributed stochastic neighbor embedding (t‐SNE) plugins that incorporated an automated learning configuration [47]. Quantitative data were analyzed and visualized using R (v3.5.1) that ran on RStudio (v2022.02.1). Principal component analysis was performed using the “stats” and “ggbiplot” packages. Normal distribution was assumed and two‐sided statistical tests were performed for all data. Paired sample means were analyzed by the paired *t*‐test. Two independent samples were compared by the *t*‐test. Analysis of variance was performed for multiple group comparisons. A *p*‐value of <0.05 was considered statistically significant.

## Author Contributions

Brigitte Müller‐Hilke supervised the study and provided resources. Johann Aleith conceptualized the study and prepared the first draft of the manuscript. Wendy Bergmann‐Ewert and Johann Aleith collected the data, analyzed and interpreted the results, and prepared the first draft of the manuscript. All authors reviewed the results and approved the final version of the manuscript.

## Conflicts of Interest

The authors declare no conflicts of interest.

### Peer Review

The peer review history for this article is available at https://publons.com/publon/10.1002/eji.202451193

## Supporting information



Supporting Information

## Data Availability

Unmixed flow cytometry data of multicolor experiments are openly available at “http://sch197.med.uni‐rostock.de/dl/DFG‐SEPRA‐IMMUN”. Raw flow cytometry data of single‐labeling experiments as well as controls will be made available by the corresponding author upon reasonable request.
