# Supplementary material for: Maximizing Insights, Minimizing Animal Testing: A Framework for Validating Multiparametric Single‐Cell Cytokine Analysis Panels
Source: Eur J Immunol. 2025 Mar 12;55(3):e202451193. doi: 10.1002/eji.202451193 (PMC11898573; doi:10.1002/eji.202451193)
Supplement: Supplementary file 1 — Supporting Information [file EJI-55-e202451193-s001.pdf]

Maximizing Insights, Minimizing Animal Testing: A Framework for Validating Multiparametric Single-Cell Cytokine Analysis Panels

Johann Aleith *et al.*

Supplementary Information

Contents:

Supplementary Figures

Supplementary Figure 1: Extraction and culture of primary cells for single antibody labeling of cell surface antigens.
Supplementary Figure 2: Single labeling of antigens using primary cells.
Supplementary Figure 3: Testing different antibody conjugate concentrations for surface antigen labeling.
Supplementary Figure 4: Testing different antibody conjugate concentrations for intracellular antigen labeling.
Supplementary Figure 5: Global expressions of surface and intracellular antigens following in vitro restimulation.
Supplementary Figure 6: TNFα expression among lymphocyte subpopulation after restimulation.
Supplementary Figure 7: Cytokine productions among myeloid cells upon restimulation.
Supplementary Figure 8: Identification of Fibroblast-like synoviocytes (FLS) by Boolean gating.
Supplementary Figure 9: Global expressions of surface and intracellular antigens in restimulated co-cultures.
Supplementary Figure 10: Cytokine expressions among lymphocyte subpopulations following restimulation of co-cultures.
Supplementary Figure 11: Cytokine expressions by double negative and $CD8^+$ T cells following restimulation.
Supplementary Figure 12: Expression of cytokines among myeloid cell populations following the restimulation of co-cultures.
Supplementary Figure 13: The immunological landscapes of spleen, peripheral blood and liver following the in vivo application of Brefeldin A.

Supplementary Tables

Supplementary Table 1: Selection of antibody-fluorophore conjugates for spectral flow cytometry.
Supplementary Table 2: Reference controls for spectral unmixing in multicolor panels.

Supplementary Figures

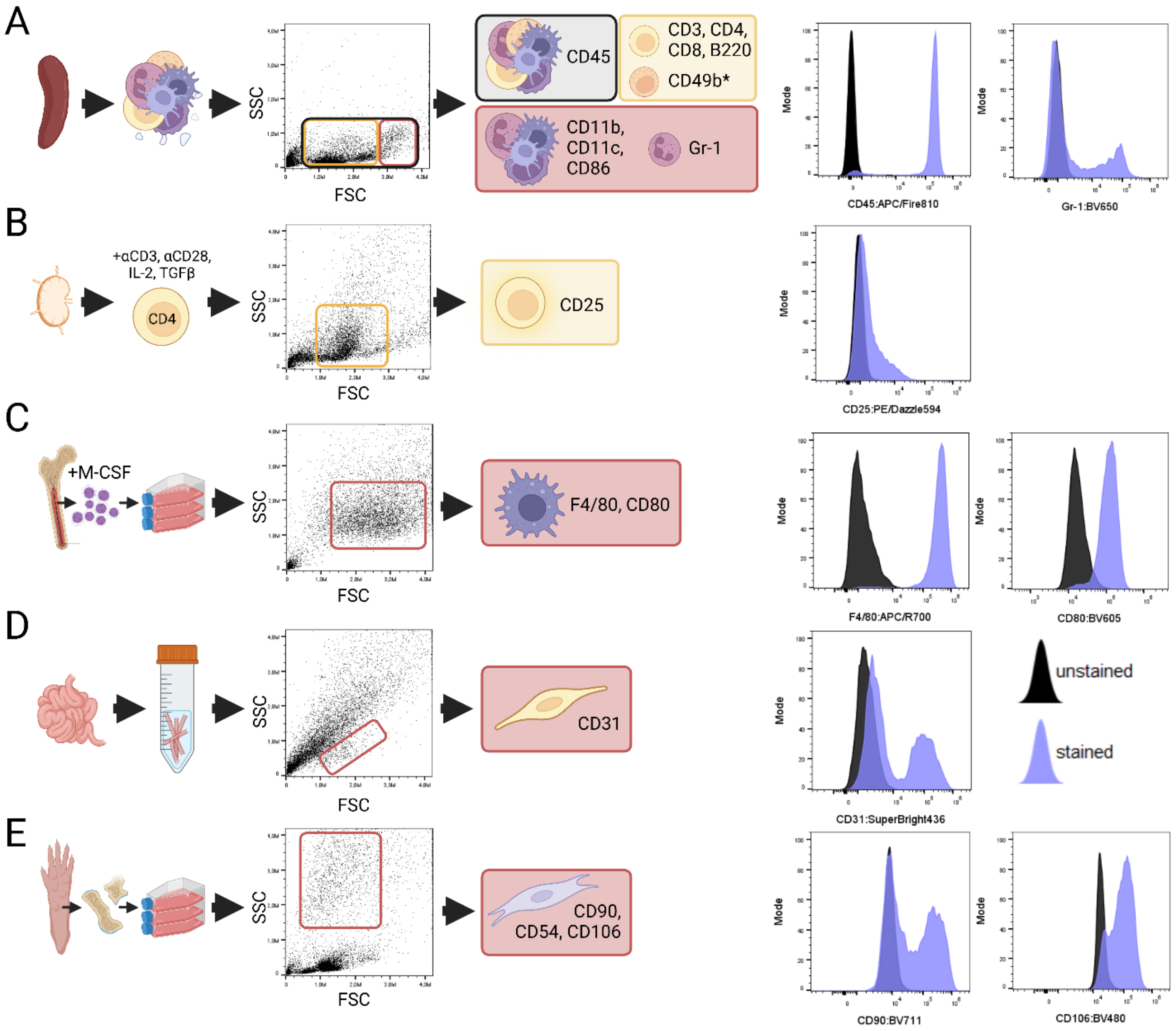

Supplementary Figure 1: Extraction and culture of primary cells for single antibody labeling of cell surface antigens. (**A**) Splenocytes were subjected to antibody labeling of common leukocyte surface antigens. Expression of CD45 was evaluated on leukocytes (black rectangle gate). CD3, CD4, CD8, B220 and CD49b were assessed on FSC^{lo} splenocytes (*i.e.* lymphocytes, yellow rectangle gate). CD11b, CD11c, CD86 and Gr-1 were analyzed on FSC^{hi} splenocytes (*i.e.* myeloid cells, red rectangle gate). Histograms show the background (auto-)fluorescence (black) and distribution of labeled cells (purple) for the displayed channels. *Labeling of CD49b was performed for Panel 1 only. (**B**) $CD4^{+}$ cells were purified from lymph nodes by magnetic cell sorting and cultured in differentiation medium containing CD3 and CD28 activating antibodies, interleukin (IL-)2 and transforming growth factor (TGF)β. Activated $CD4^{+}$ T cells were then used for the labeling of CD25. (**C**) Bone marrow cells were obtained from long bones and differentiated using macrophage colony-stimulating factor (M-CSF). Bone marrow-derived macrophages were subsequently used for labeling of F4/80 and CD80, respectively. (**D**) Endothelial cells were extracted from small intestines by enzymatic digestion and were used for CD31 labeling. (**E**) CD90, CD54 and CD106 were assessed on fibroblast-like synoviocytes that were obtained from cultures following enzymatic digestion of paws.

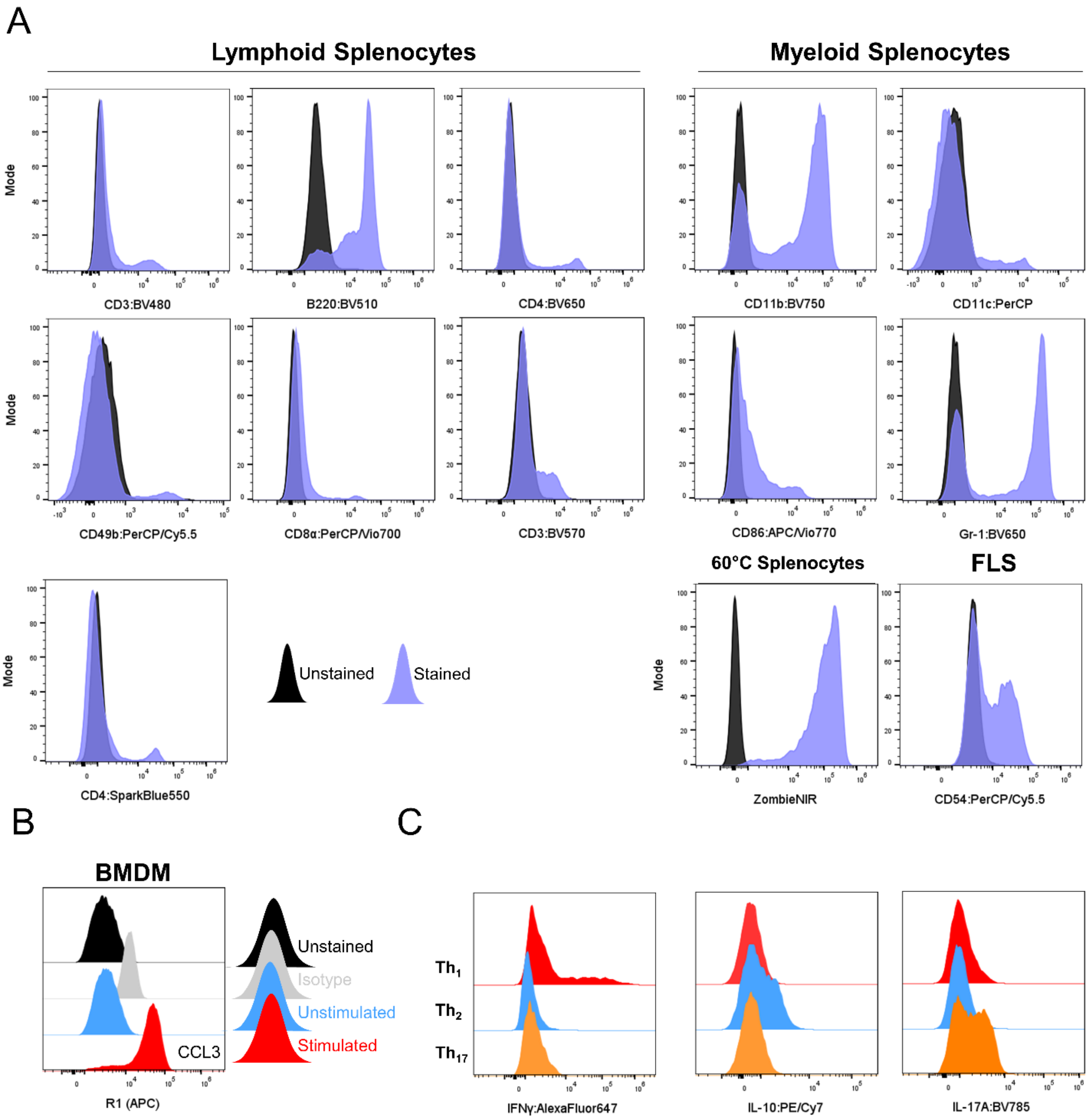

Supplementary Figure 2: Single labeling of antigens using primary cells. (**A**) Using manual gating, lymphoid and myeloid splenocytes were identified as FSC^{lo} and FSC^{hi} cells, respectively. Single labeling of CD3, CD4, CD8, B220 and CD49b was assessed on lymphoid splenocytes. Expression of CD11b, CD11c, CD86 and Gr-1 were evaluated on myeloid splenocytes. For the assessment of dead cell labeling, splenocytes were incubated at 60°C and then subjected to ZombieNIR staining. CD54 expression was analyzed on Fibroblast-like synoviocytes (FLS). (**B**) Bone marrow-derived macrophages (BMDM) were used for the intracellular labeling of CCL3. Histograms display the background fluorescence (black) for the indicated channels, along with fluorescence intensities following labeling with isotype controls (grey) and labeling with test antibodies applied to unstimulated cells (blue) or stimulated cells (red). (**C**) Cross-labeling of in vitro-polarized and PMA/ionomycin-stimulated type 1 helper T cells (Th_1, red), Th_2 (blue) and Th_{17} (orange) with IFNγ, IL-10 and IL-17A

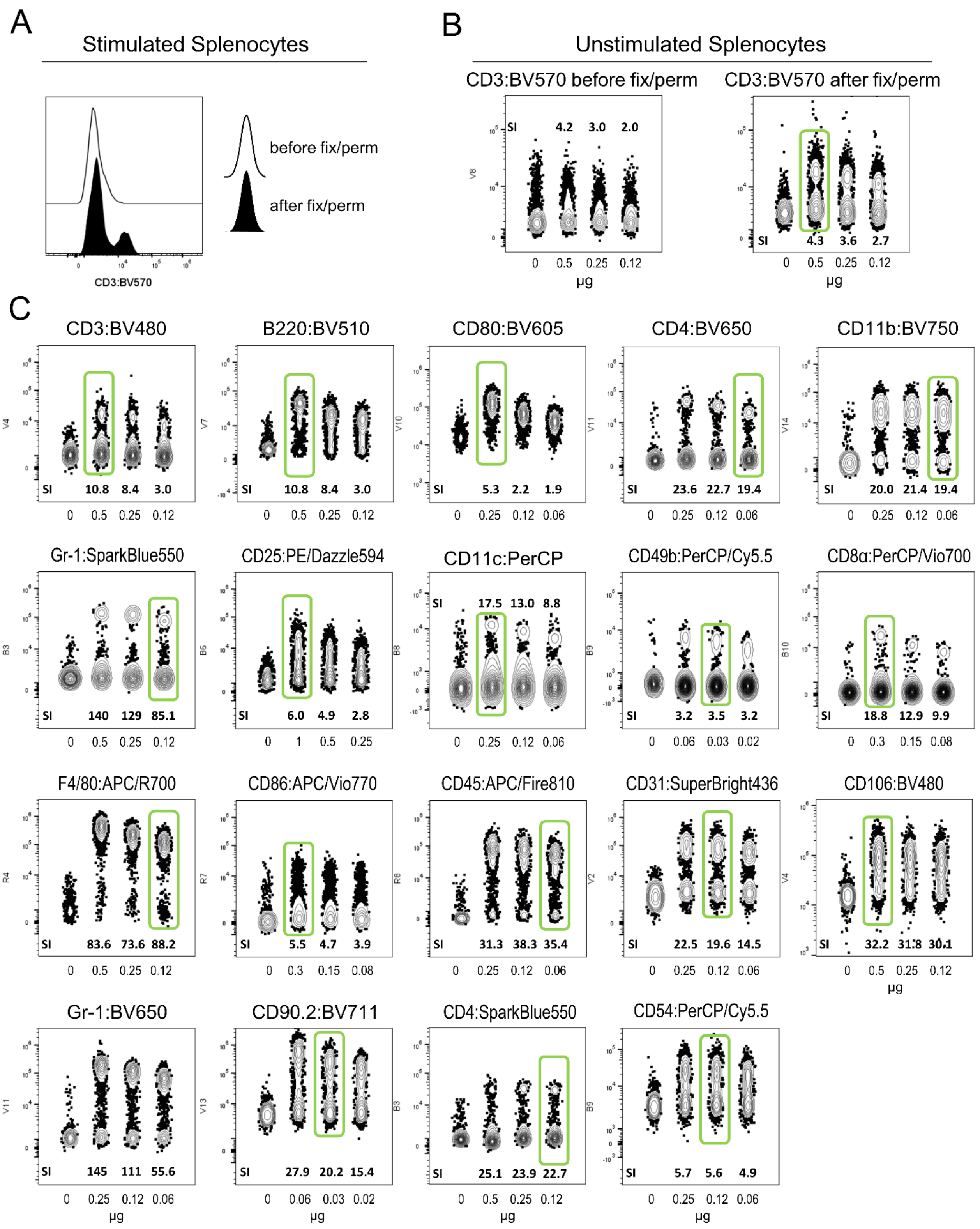

A
Stimulated Splenocytes
before fix/perm
after fix/perm
CD3:BV570
B
Unstimulated Splenocytes
CD3:BV570 before fix/perm
SI 4.2 3.0 2.0
0 0.5 0.25 0.12
µg
CD3:BV570 after fix/perm
SI 4.3 3.6 2.7
0 0.5 0.25 0.12
µg
C
CD3:BV480
SI 10.8 8.4 3.0
0 0.5 0.25 0.12
B220:BV510
SI 10.8 8.4 3.0
0 0.5 0.25 0.12
CD80:BV605
SI 5.3 2.2 1.9
0 0.25 0.12 0.06
CD4:BV650
SI 23.6 22.7 19.4
0 0.25 0.12 0.06
CD11b:BV750
SI 20.0 21.4 19.4
0 0.25 0.12 0.06
Gr-1:SparkBlue550
SI 140 129 85.1
0 0.5 0.25 0.12
CD25:PE/Dazzle594
SI 6.0 4.9 2.8
0 1 0.5 0.25
CD11c:PerCP
SI 17.5 13.0 8.8
0 0.25 0.12 0.06
CD49b:PerCP/Cy5.5
SI 3.2 3.5 3.2
0 0.06 0.03 0.02
CD8α:PerCP/Vio700
SI 18.8 12.9 9.9
0 0.3 0.15 0.08
F4/80:APC/R700
SI 83.6 73.6 88.2
0 0.5 0.25 0.12
CD86:APC/Vio770
SI 5.5 4.7 3.9
0 0.3 0.15 0.08
CD45:APC/Fire810
SI 31.3 38.3 35.4
0 0.25 0.12 0.06
CD31:SuperBright436
SI 22.5 19.6 14.5
0 0.25 0.12 0.06
CD106:BV480
SI 32.2 31.8 30.1
0 0.5 0.25 0.12
µg
Gr-1:BV650
SI 145 111 55.6
0 0.25 0.12 0.06
µg
CD90.2:BV711
SI 27.9 20.2 15.4
0 0.06 0.03 0.02
µg
CD4:SparkBlue550
SI 25.1 23.9 22.7
0 0.5 0.25 0.12
µg
CD54:PerCP/Cy5.5
SI 5.7 5.6 4.9
0 0.25 0.12 0.06
µg

Supplementary Figure 3: Testing different antibody conjugate concentrations for surface antigen labeling. (**A**) Splenocytes were stimulated with PMA and ionomycin. Histograms show the labeling efficiency for CD3:BV570 either before (white) or after fixation/permeablilization (black). (**B**) Antibody conjugate titration results for unstimulated splenocytes. Serial contour plots display labeling resolutions of CD3:BV570 either before (left) or after fixation/permeablilization (right). Green rectangles illustrate conjugate amounts that were selected for later multicolor experiments. (**C**) Antibody conjugate titration results for surface antigens constitutively expressed on splenocytes (CD3, B220, CD4, CD11b, Gr-1, CD11c, CD49b,CD8α, CD86, CD45), bone marrow-derive macrophages (CD80, F4/80), activated T cell (CD25), instestinal epithelial cells (CD31) and fibroblast-like synoviocytes (CD106, CD90, CD54). SI: Staining Index.

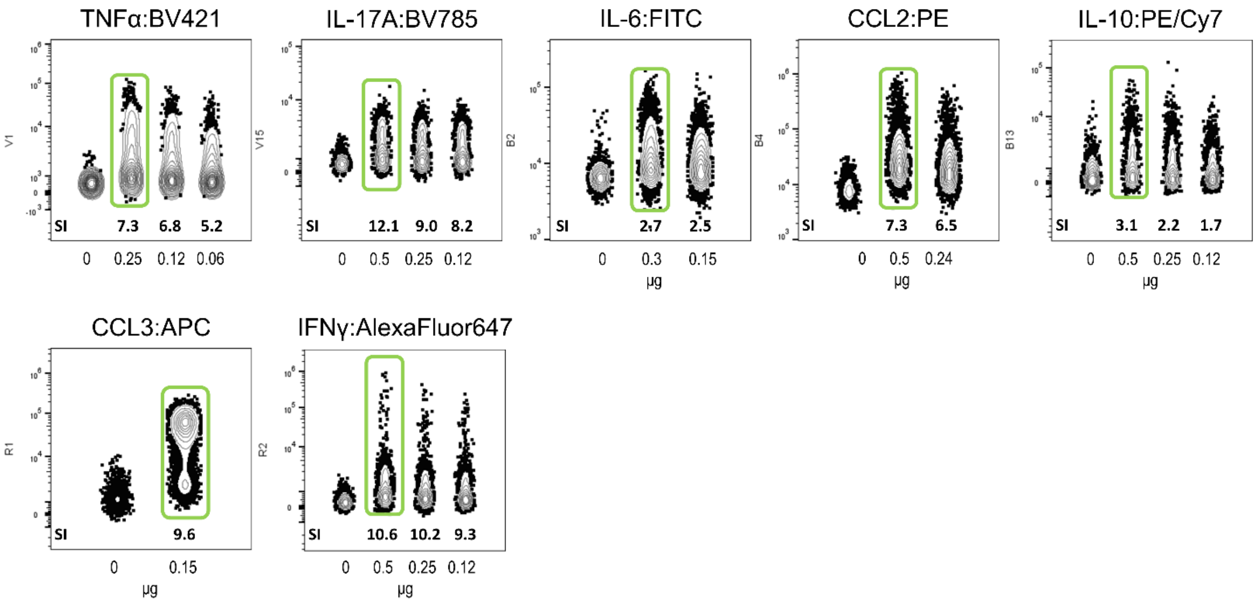

Supplementary Figure 4: Testing different antibody conjugate concentrations for intracellular antigen labeling. Serial contour plots display labeling resolutions. Cytokine expression were induced by in vitro stimulation of splenocytes (TNFα), type 1 helper T cells (Th_1, IFNγ), Th_2 cell (IL-10), Th_{17} cell (IL-17A) and bone marrow-derived macrophages (IL-6, CCL2, CCL3). Green rectangles illustrate conjugate amounts that were selected for later multicolor experiments. SI: Staining Index

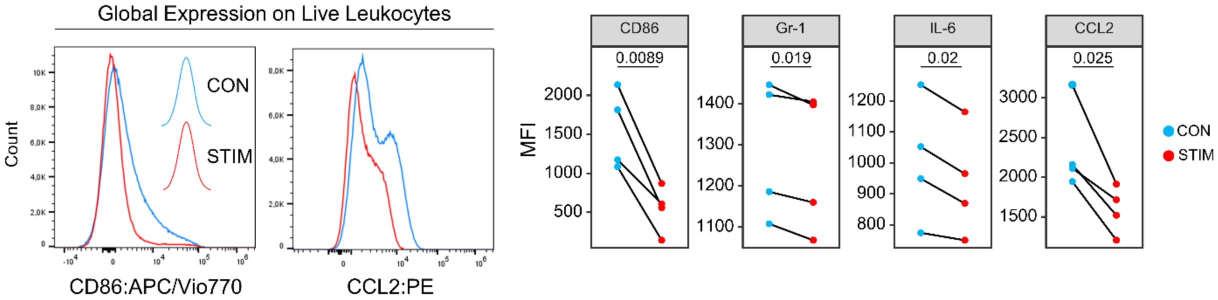

Supplementary Figure 5: Global expressions of surface and intracellular antigens following in vitro restimulation. In vitro-polarized Th cells and freshly isolated splenocytes were restimulated separately followed by conjoined multicolor labeling and flow cytometry analysis. Data was generated from two independent experiments. Representative histograms show the fluorescence intensity among live leukocytes for the labeling of CD86 and CCL2 when comparing cells from a unstimulated sample (CON) to cells from a stimulated sample (STIM). Dot plots show quantitative analyses of global expression of CD86, Gr-1, IL-6 and CCL2. P values resulted from paired t tests.

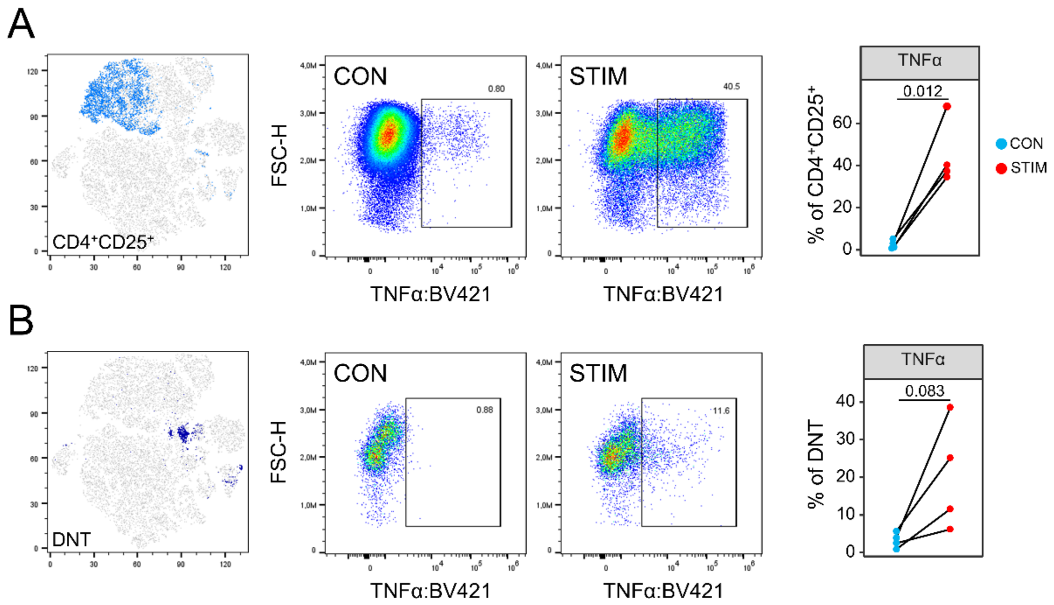

Supplementary Figure 6: TNFα expression among lymphocyte subpopulation after restimulation. In vitro-polarized Th cells and freshly isolated splenocytes were restimulated separately followed by conjoined multicolor labeling and flow cytometry analysis. Data was generated from two independent experiments. Representative pseudocolor plots and dot plots show the proportion of cells that expressed TNFα among $CD4^+$ effector T cells ($CD4_{eff}$, **A**) and CD4/CD8 double negative T cells (DNT, **B**), respectively. CON: non-stimulated cultures. STIM: stimulated cultures. P values resulted from paired t tests.

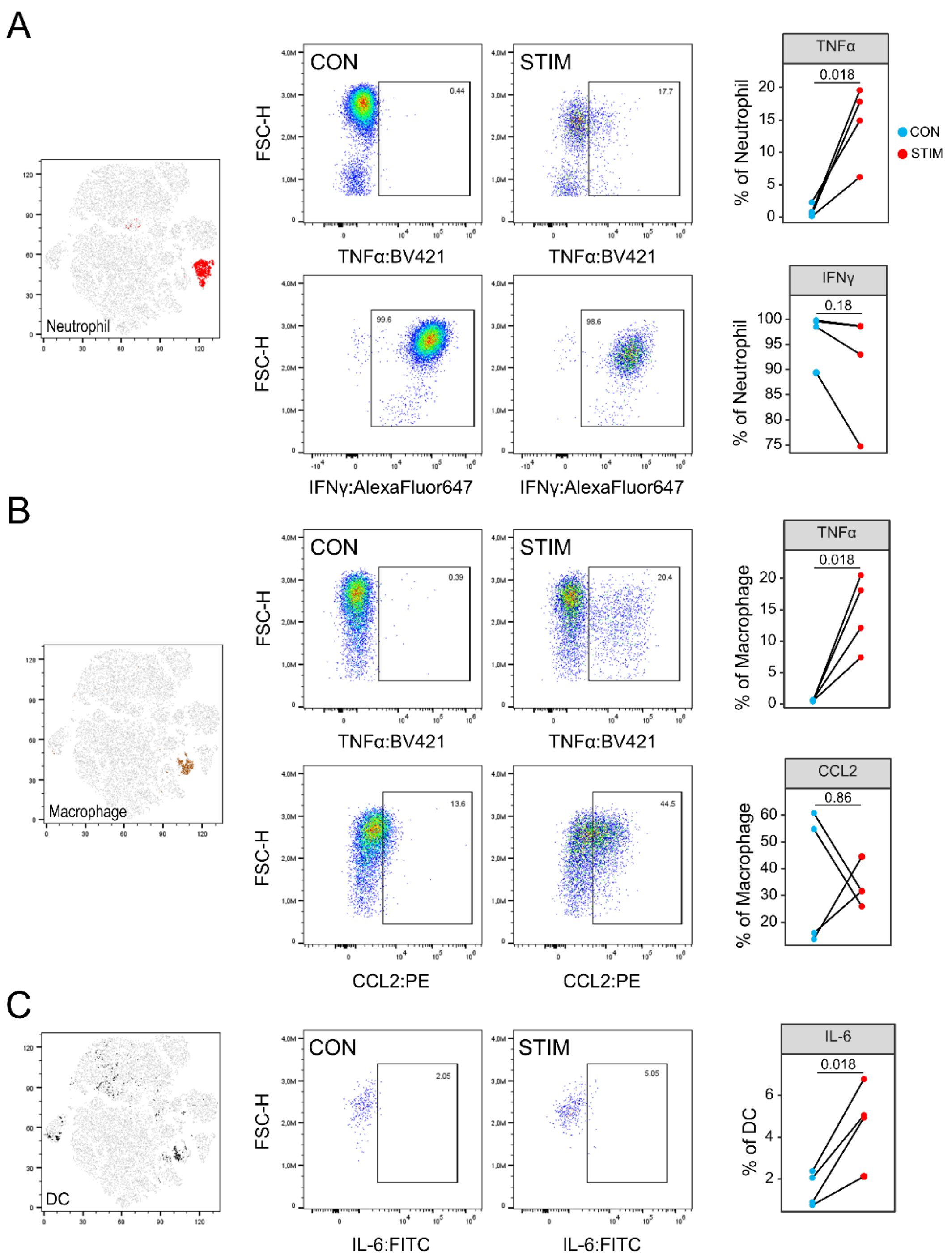

Supplementary Figure 7: Cytokine productions among myeloid cells upon restimulation. In vitro-polarized Th cells and freshly isolated splenocytes were restimulated separately followed by conjoined multicolor labeling and flow cytometry analysis. Data was generated from two independent experiments. (**A**) Representative pseudocolor and dot plots show the proportions of neutrophils that expressed TNFα and IFNγ. (**B**) Representative pseudocolor plots and dot plots show the proportions of macrophages that expressed TNFα and CCL2. (**C**) Flow cytometry data show the percentage of DCs that expressed IL-6. CON: non-stimulated cultures. STIM: stimulated cultures. P values resulted from paired t tests.

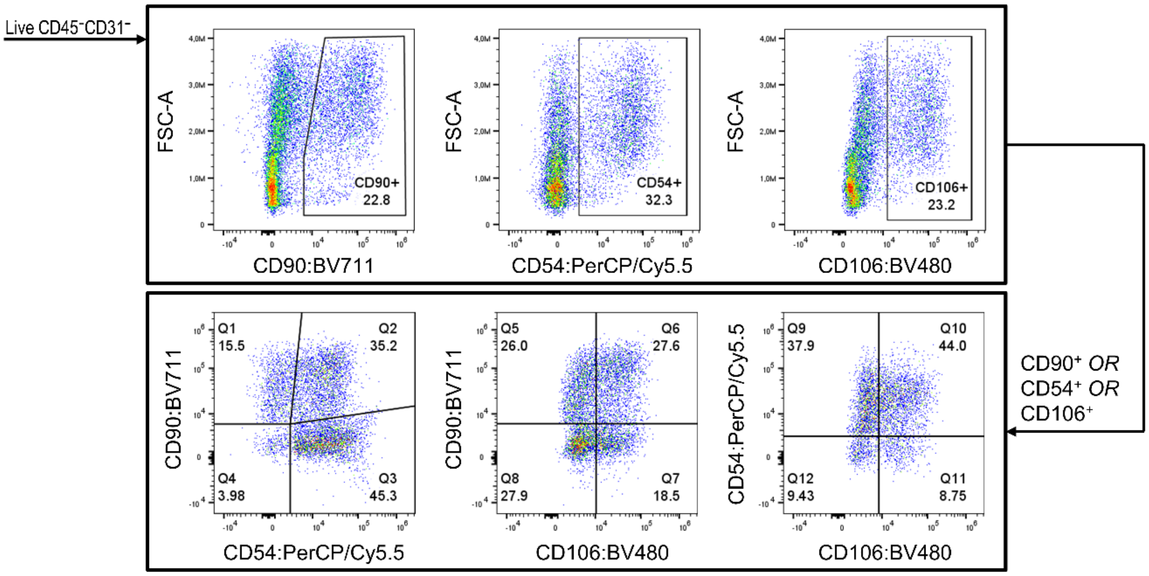

Supplementary Figure 8: Identification of Fibroblast-like synoviocytes (FLS) by Boolean gating. Samples were generated from co-cultures of helper T cells, splenocytes and FLS. Representative pseudocolor plots show the gating scheme of CD90⁺, CD54⁺ and CD106⁺ cells among live CD45⁻CD31⁻ singlets (top). FLS were identified as a population that expressed any given combination of these surface antigens. Co-expression of CD90, CD54 and CD106 was verified for the classification of FLS subpopulations (bottom).

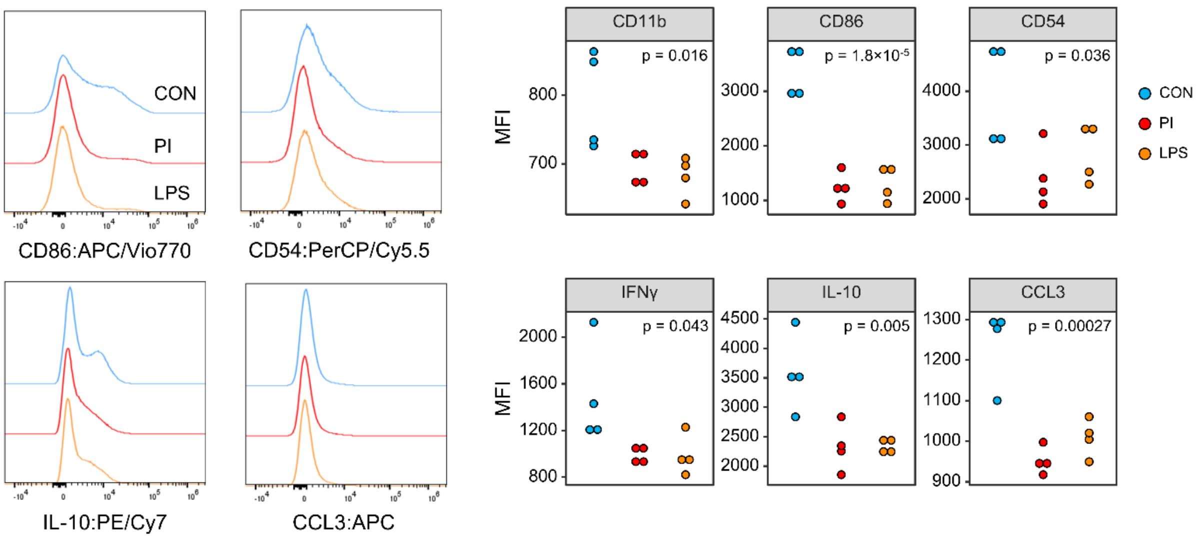

Supplementary Figure 9: Global expressions of surface and intracellular antigens in restimulated co-cultures. In vitro-polarized Th cells, Fibroblast-like synocioytes (FLS) and freshly isolated splenocytes were restimulated followed by multicolor labeling and flow cytometry analysis. Data was generated from two independent experiments. Representative histograms show fluorescence intensities of CD86, CD54, IL-10 and CCL3. Dotplots show quantitative expression data of live single cells based on median fluorescence intensities (MFI). CON: non-stimulated co-cultures. PI: co-cultures restimulated with PMA/ionomycin. LPS: co-cultures restimulated with LPS. P values resulted from one-way ANOVAs.

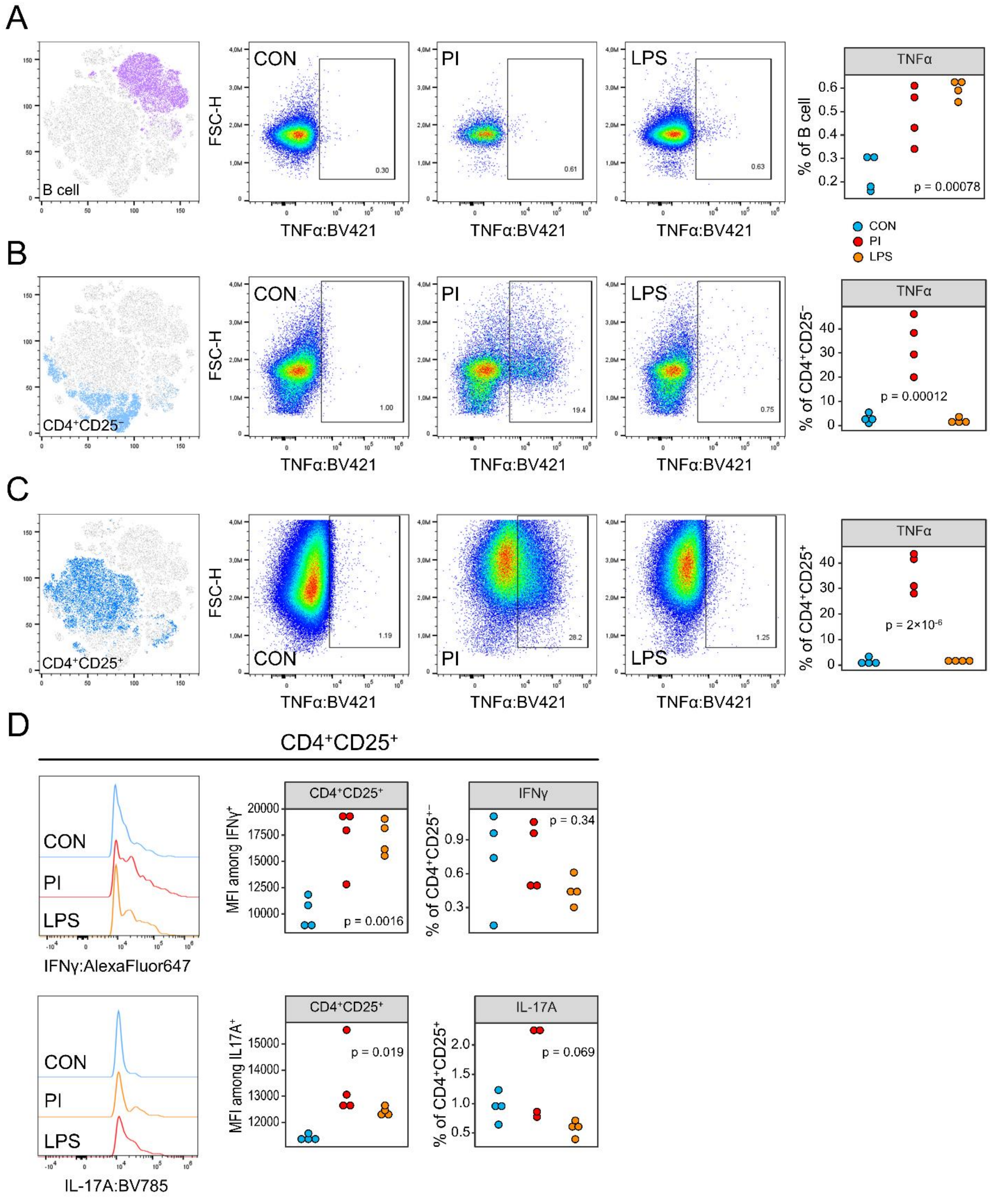
A
B cell
CON
PI
LPS
FSC-H
TNFα:BV421
0.30
0.61
0.63
TNFα
% of B cell
p = 0.00078
CON
PI
LPS
B
CD4+CD25−
1.00
19.4
0.75
% of CD4+CD25−
p = 0.00012
C
CD4+CD25+
1.19
28.2
1.25
% of CD4+CD25+
p = 2×10-6
D
CD4+CD25+
IFNγ:AlexaFluor647
MFI among IFNγ+
p = 0.0016
IFNγ
p = 0.34
IL-17A:BV785
MFI among IL17A+
p = 0.019
IL-17A
p = 0.069

Supplementary Figure 10: Cytokine expressions among lymphocyte subpopulations following restimulation of co-cultures. In vitro-polarized Th cells, Fibroblast-like synocioytes (FLS) and freshly isolated splenocytes were restimulated followed by multicolor labeling and flow cytometry analysis. Data was generated from two independent experiments. (**A**-**C**) Representative pseudocolor plots and dot plots show the proportion of cells that expressed TNFα cells among B cells (A), CD4$^+$ naïve T cells (B) and CD4$^+$ effector T cells (C). (**D**) Flow cytometry data show the expressions of IFNγ (top) and IL-17A (bottom) in CD4$^+$ effector T cells based on median fluorescence intensities (MFI). CON: non-stimulated co-cultures. PI: co-cultures restimulated with PMA/ionomycin. LPS: co-cultures restimulated with LPS. P values resulted from one-way ANOVAs.

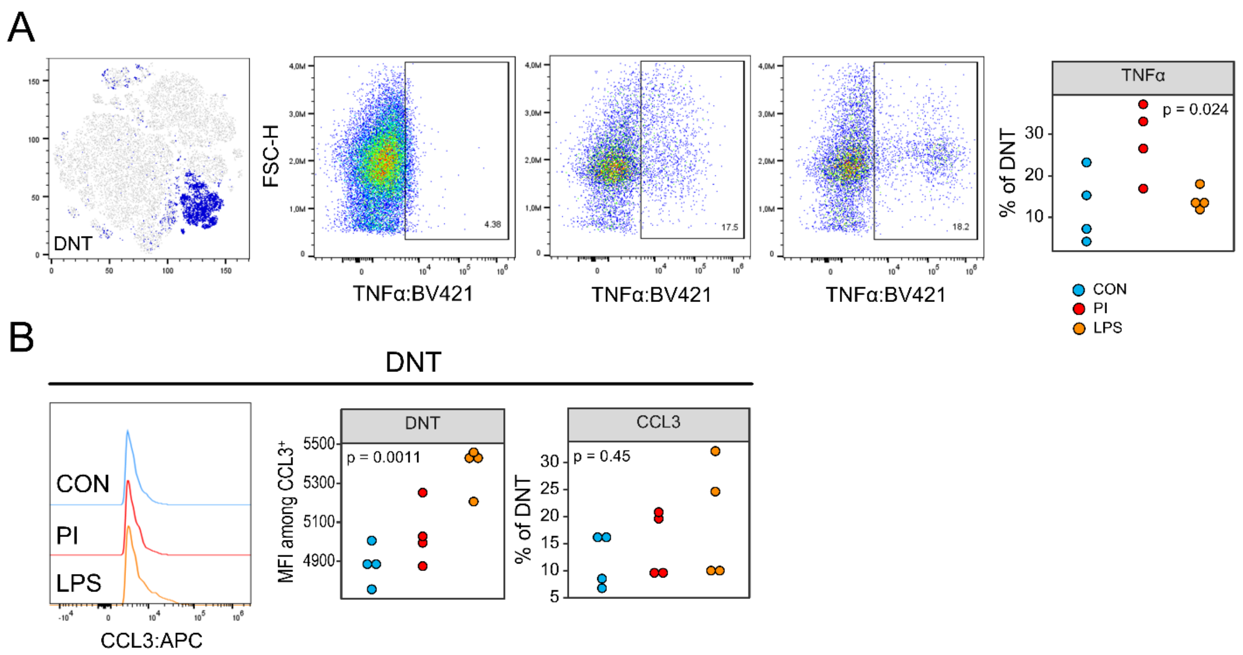

Supplementary Figure 11: Cytokine expressions by double negative and CD8$^+$ T cells following restimulation. In vitro-polarized Th cells, Fibroblast-like synocioytes (FLS) and freshly isolated splenocytes were restimulated followed by multicolor labeling and flow cytometry analysis. Data was generated from two independent experiments. (**A**) Flow cytometry data show the proportions of CD4/CD8 double negative T cells (DNTs) that expressed TNFα. (**B**) Representative histograms and quantitative data show the epxression of CCL3 in DNTs based on median fluorescence intensities (MFI). CON: non-stimulated co-cultures. PI: co-cultures restimulated with PMA/ionomycin. LPS: co-cultures restimulated with LPS. P values resulted from one-way ANOVAs.

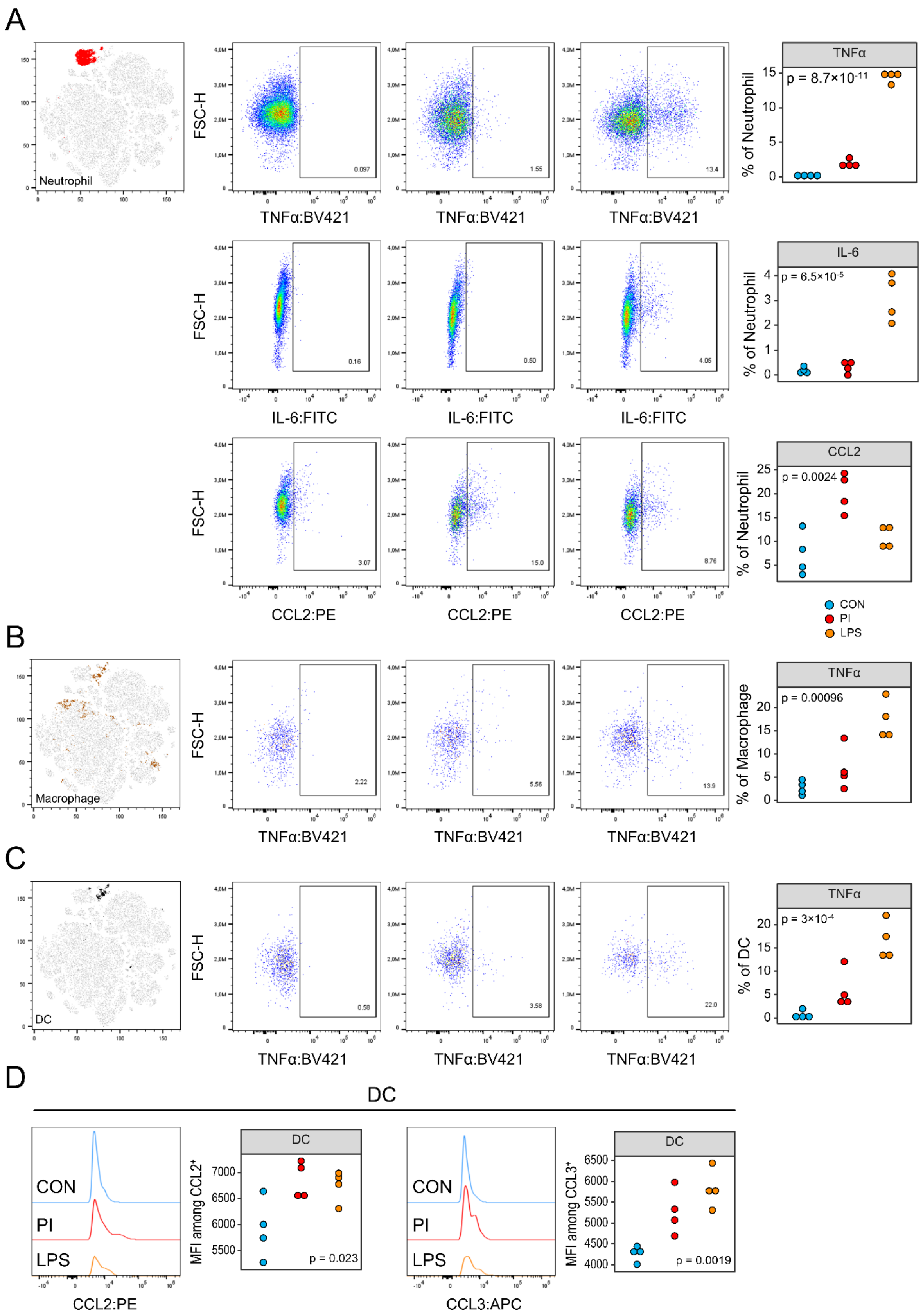

A
Neutrophil
FSC-H
TNFα:BV421
0.097
1.55
13.4
TNFα
p = 8.7×10-11
% of Neutrophil
IL-6:FITC
0.16
0.50
4.05
IL-6
p = 6.5×10-5
CCL2:PE
3.07
15.0
8.76
CCL2
p = 0.0024
CON
PI
LPS
B
Macrophage
2.22
5.56
13.9
p = 0.00096
% of Macrophage
C
DC
0.58
3.58
22.0
p = 3×10-4
% of DC
D
DC
CON
PI
LPS
CCL2:PE
MFI among CCL2+
p = 0.023
CCL3:APC
MFI among CCL3+
p = 0.0019

Supplementary Figure 12: Expression of cytokines among myeloid cell populations following the restimulation of co-cultures. In vitro-polarized Th cells, Fibroblast-like synocioytes (FLS) and freshly isolated splenocytes were restimulated followed by multicolor labeling and flow cytometry analysis. Data was generated from two independent experiments. (**A**) Representative pseudocolor plots and quantitative flow cytometry data show the proportions of neutrophils that expressed TNFα, IL-6 and CCL2. (**B**-**C**) Representative pseudocolor plots and quantitative flow cytometry data show the proportions of macrophages (B) and DCs (C) that expressed TNFα. (**D**) Flow cytometry data illustrate CCL3 expressions among DCs based on median fluorescence intensities (MFI). CON: non-stimulated co-cultures. PI: co-cultures restimulated with PMA/ionomycin. LPS: co-cultures restimulated with LPS. P values resulted from one-way ANOVAs.

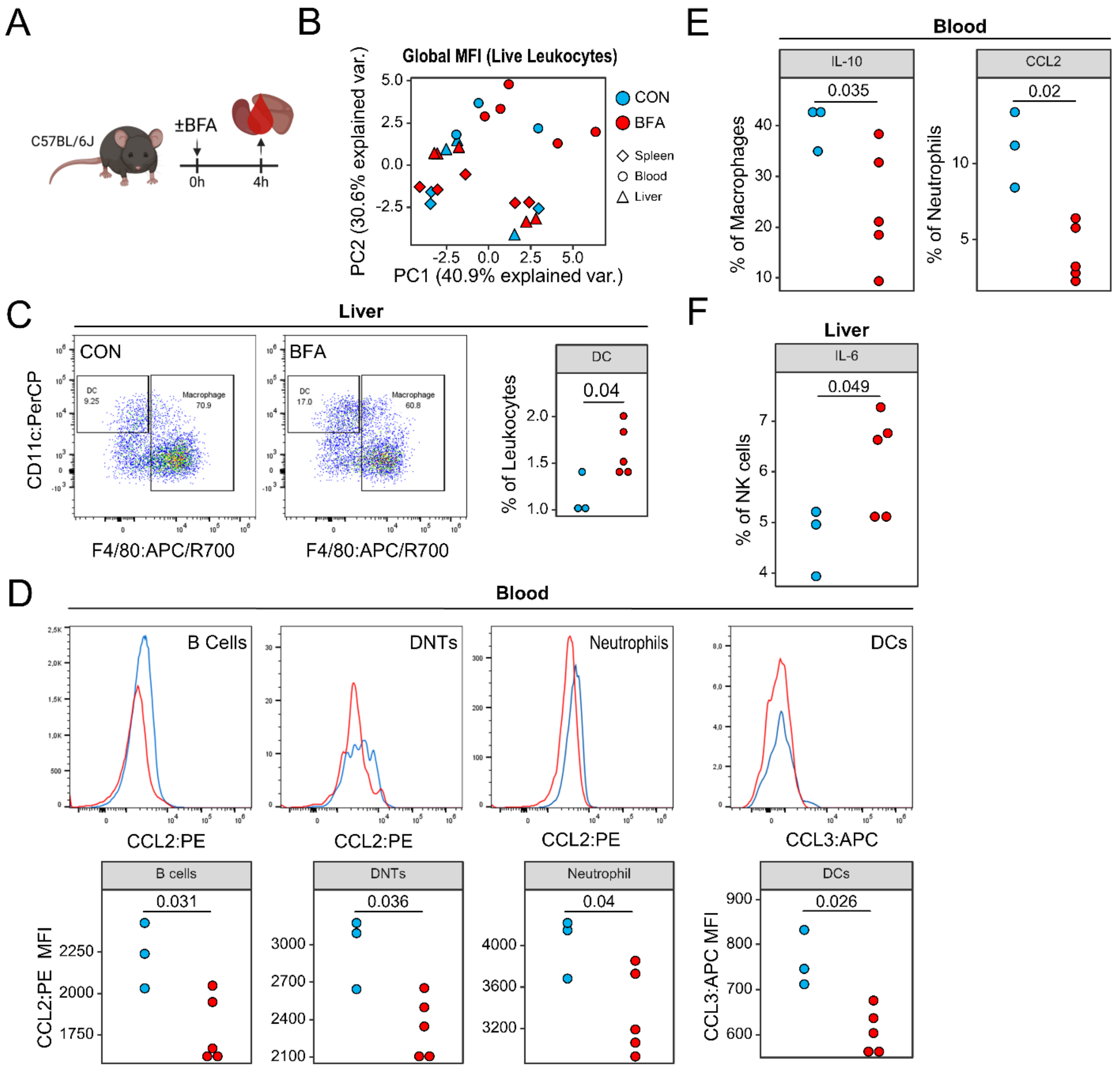

Supplementary Figure 13: The immunological landscapes of spleen, peripheral blood and liver following the in vivo application of Brefeldin A. (**A**) Experimental scheme. Six- to eight-week-old C57BL/6J mice were either intraperitoneally injected with 10 mg/kg Brefeldin (BFA, n = 5) or left untreated (CON, n = 3). Mice were monitored for four hours and then sacrificed for the collections of spleen, blood and liver. (**B**) Visualization of principal component analysis based on global expression values among leukocytes. (**C**) Representative pseudocolor plots and the dot plot show the proportions of liver DCs among living leukocytes. (**D**) Representative histograms (top) and dot plots (bottom) show the expressions of CCL2 among B cells, DNTs and Neutrophils as well as the expression of CCL3 among DCs based on median fluorescence intensities (MFI). (**E**) Dot plots show the proportions of macrophages and neutrophils that expressed IL-10 and CCL2, respectively. (**F**) The dot plot shows the percentage of NK cells that expressed IL-6.

Supplementary Tables

Supplementary Table 1: Selection of antibody-fluorophore conjugates for spectral flow cytometry.

Laser ($\lambda_{exciation}$)	Detector	Antibody:Fluorophore (Clone) Panel 1	Antibody:Fluorophore (Clone) Panel 2
Violet (405 nm)	V1	TNFα:BV421 (MP6-XT22)	TNFα:BV421 (MP6-XT22)
	V2	Ø	CD31:Superbright436 (390)
	V4	CD3:BV480 (17A2)	CD106:BV480 (429)
	V7	B220:BV510 (RA3-6B2)	B220:BV510 (RA3-6B2)
	V8	Ø	CD3:BV570 (17A2)
	V10	CD80:BV605 (16-10A1)	CD80:BV605 (16-10A1)
	V11	CD4:BV650 (GK1.5)	Gr-1:BV650 (RB6-8C5)
	V13	Ø	CD90.2:BV711 (30-H12)
	V14	CD11b:BV750 (M1/70)	CD11b:BV750 (M1/70)
	V15	IL-17A:BV785 (TC11-18H10.1)	IL-17A:BV785 (TC11-18H10.1)
Blue (488 nm)	B2	IL-6:FITC (REA1034)	IL-6:FITC (REA1034)
	B3	Gr-1:SparkBlue550 (RB6-8C5)	CD4:SparkBlue550 (GK1.5)
	B4	CCL2:PE (2H5)	CCL2:PE (2H5)
	B6	CD25:PE/Dazzle594 (3C7)	CD25:PE/Dazzle594 (3C7)
	B8	CD11c:PerCP (N418)	CD11c:PerCP (N418)
	B9	CD49b:PerCP/Cy5.5 (HMα2)	CD54:PerCP/Cy5.5 (YN1/1.7.4)
	B10	CD8α:PerCP/Vio700 (REA601)	CD8α:PerCP/Vio700 (REA601)
	B13	IL-10:PE/Cy7 (JES5-16E3)	IL-10:PE/Cy7 (JES5-16E3)
Red (633 nm)	R1	CCL3:APC (REA355)	CCL3:APC (REA355)
	R2	IFNγ:AlexaFluor647 (XMG1.2)	IFNγ:AlexaFluor647 (XMG1.2)
	R4	F4/80:APC/R700 (T45-2342)	F4/80:APC/R700 (T45-2342)
	R6	ZombieNIR (Dead Cell Discriminator)	ZombieNIR (Dead Cell Discriminator)
	R7	CD86:APC/Vio770 (PO3.3)	CD86:APC/Vio770 (PO3.3)
	R8	CD45:APC/Fire810 (30-F11)	CD45:APC/Fire810 (30-F11)

Ø: Detector was not assigned in this panel

Supplementary Table 2: Reference controls for spectral unmixing in multicolor panels.

Sample type for unstained and reference control[1]	Panel	Antibody:Fluorophore
FSC^{lo} (*i.e.* lymphoid) cells from spleen	1	CD3:BV480
		CD4:BV650
		CD49b:PerCP/Cy5.5
	1 & 2	B220:BV510
		CD8α:PerCP/Vio700
	2	CD4:SparkBlue550
		CD3:BV570[2]
FSC^{hi} (*i.e.* myeloid) cells from spleen	1	Gr-1:SparkBlue550
	1 & 2	CD11b:BV750
		CD11c:PerCP
		CD86:APC/Vio770
	2	Gr-1:BV650
FSC^{hi} cells from PMA/Ionomycin stimulated splenocytes	1 & 2	TNFα:BV421[2]
Leukocytes from spleen	1 & 2	CD45:APC/Fire810
Activated T cell culture	1 & 2	CD25:PE/Dazzle594
Cells from bone marrow-derived macrophage culture	1 & 2	CD80:BV605
		F4/80:APC/R700
SSC^{lo} (*i.e.* endothelial) cells from small intestine digestion	2	CD31:SuperBright436
Cells from fibroblast-like synoviocytes culture	2	CD106:BV480
		CD90.2:BV711
		CD54:PerCP/Cy5.5
Beads	1 & 2	IL-17A:BV785[2]
		IL-6:FITC[2,3]
		CCL2:PE[2]
		IL-10:PE/Cy7[2]
		CCL3:APC[2,3]
		IFNγ:AlexaFluor647[2]

[1]See Supplementary Figure 1 & Figure 1 (main text) for gating, [2]Fluorochromes used for intracellular staining were not exposed to formaldehyde and saponines, [3]Beads capable of binding to antibodies from REAfinity clones
